# Advanced Biomaterials for Cell‐Specific Modulation and Restore of Cancer Immunotherapy

**DOI:** 10.1002/advs.202200027

**Published:** 2022-03-27

**Authors:** Shaobo Ruan, Yuanyu Huang, Mei He, Huile Gao

**Affiliations:** ^1^ Advanced Research Institute of Multidisciplinary Science Beijing Institute of Technology Beijing 100081 China; ^2^ College of Pharmacy University of Florida Gainesville FL 32610 USA; ^3^ West China School of Pharmacy Sichuan University Chengdu 610041 China

**Keywords:** biomaterials, cancer immunotherapy, drug delivery, targeting modulation, vaccines

## Abstract

The past decade has witnessed the explosive development of cancer immunotherapies. Nevertheless, low immunogenicity, limited specificity, poor delivery efficiency, and off‐target side effects remain to be the major limitations for broad implementation of cancer immunotherapies to patient bedside. Encouragingly, advanced biomaterials offering cell‐specific modulation of immunological cues bring new solutions for improving the therapeutic efficacy while relieving side effect risks. In this review, focus is given on how functional biomaterials can enable cell‐specific modulation of cancer immunotherapy within the cancer‐immune cycle, with particular emphasis on antigen‐presenting cells (APCs), T cells, and tumor microenvironment (TME)‐resident cells. By reviewing the current progress in biomaterial‐based cancer immunotherapy, here the aim is to provide a better understanding of biomaterials’ role in targeting modulation of antitumor immunity step‐by‐step and guidelines for rationally developing targeting biomaterials for more personalized cancer immunotherapy. Moreover, the current challenge and future perspective regarding the potential application and clinical translation will also be discussed.

## Graphical abstract

This review mainly focuses on how biomaterials realize cell‐targeted modulation of antitumor immunity by dissecting their roles into three parts: 1) targeted modulation of antigen‐presenting cells for enhancing vaccine efficiency; 2) targeted modulation of T cells both ex vivo and in vivo for expansion and persistent function; and 3) targeted modulation of tumor microenvironment (TME)‐resident cells for reversing immunosuppressive TME.

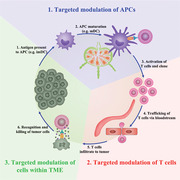

## 1 Introduction

Cancer immunotherapy harnessing the power of body's own immune system to kill cancer cell has been witnessed to achieve great success in both preclinical and clinical trials in the past decades.^[^ ^1^ ^]^ Unlike post‐surgery radiation therapy and chemotherapy which often lead to serious side‐effects to patients’ normal tissue and cells, cancer immunotherapy may enable more precise killing by effector T cell's homing to tumor cells with specific antigen.^[^ ^2^ ^]^ To date, a variety of cancer immunotherapies including cytokine therapy,^[^ ^3^ ^]^ cancer vaccine,^[^ ^4^ ^]^ oncolytic viruses^[^ ^5^ ^]^ as well as many other in the pipeline have been well studied and approved by the Food and Drug Administration (FDA) for the treatment of various cancer.^[^ ^6^ ^]^ More recently, the emergence of immune checkpoint blockade (ICB) therapy, such as anti‐cytotoxic T lymphocyte antigen 4 (CLTA4),^[^ ^7^ ^]^ anti‐programmed cell death 1 (PD1)^[^ ^8^ ^]^ and anti‐programmed cell death ligand 1 (PD‐L1)^[^ ^9^ ^]^ as well as cell‐based therapy, such as chimeric antigen receptor T‐cell (CAR‐T)^[^ ^10^ ^]^ further enrich the immunotherapeutic options and regimens. ICB therapy is designed to release the “immunosuppressive brake” for the cytotoxic T lymphocytes (CTLs) to improve the infiltration of T cells into tumor and to enhance killing of targeted cancer cells.^[^ ^11^ ^]^ In CAR‐T cell therapies, the isolated T cells are genetically engineered to express CARs that recognize cognate antigen‐specific tumor cells and expanded ex vivo, and finally re‐administrated back into the same patient for tumor cell killing.^[^ ^10^, ^12^ ^]^ More importantly, the remarkable treatment benefits of ICB therapy and CAR‐T therapy greatly boosted the enthusiasm on cancer immunotherapy and motivated a massive effort to further explore more efficient therapy strategy. Although cancer immunotherapies have demonstrated great potential in combating cancer and more options are available currently, only a fraction of patients benefited from these immunotherapies and most patients showed limited response.^[^ ^13^ ^]^ Meanwhile, patients who received these treatments have to suffer from severe immune related adverse effects (irAEs), such as ICB‐related inflammatory side effects^[^ ^14^ ^]^ and CAR‐T‐related cytokine release syndrome.^[^ ^15^ ^]^ The insufficient immune response and irAEs remain to be the major challenges for wide implementation in clinical treatment, which is mainly owing to low immunogenicity, limited specificity, poor delivery efficiency, and off‐target distribution. For instance, rapid elimination, degradation, and insufficient internalization of cancer vaccines (e.g., antigenic proteins, peptides, DNA) by APCs are often observed during the delivery process, resulting in modest antitumor immune responses.^[^ ^16^ ^]^ Therefore, strategies to precisely modulate specific immune cells or tumor cells are imperative which enables a broader range of patients to be beneficial with precise antitumor immunity while much less off‐target toxicity.

Over the past decades, biomaterials, including nanoparticles (NPs),^[^ ^17^ ^]^ injectable hydrogel,^[^ ^18^ ^]^ microneedles (MNs),^[^ ^19^ ^]^ and implantable scaffolds^[^ ^20^ ^]^ have been widely explored as valuable resource to troubleshoot these issues and support this goal. As a delivery platform, biomaterials offer several superiorities over the therapeutic agents alone.^[^ ^21^ ^]^ First, biomaterials can protect therapeutic cargos from degradation post administration, prolong circulation time, and improve pharmacokinetic performance during delivery process. Second, by rationally designing or functionalizing, biomaterials can deliver immunotherapeutic cargos specifically to targeted cells within immunologic organs or TME through systemic delivery or localized delivery. In some case, they can enable dual targeting delivery to immune cells and tumor cells simultaneously. Third, these functionalized biomaterials also promote the cellular internalization of therapeutic and enable spatiotemporal control over the cargo release in response to specific stimulus intracellularly. Fourth, biomaterials such as NPs can be developed for systemic delivery of therapeutic cargos, while implants or scaffolds can be developed for local delivery. Moreover, biomaterials can enable the combination of multiple therapeutic regimens, including but not limited to immunotherapeutic agents, such as antigen with adjuvants,^[^ ^22^ ^]^ ICB with chemotherapy,^[^ ^23^ ^]^ and ICB with chemo‐photodynamic therapy.^[^ ^24^ ^]^ Therefore, compared to immunotherapeutic agents alone, biomaterials‐based cancer immunotherapies enable more precise and improved antitumor immunity whereas much reduced irAEs by circumventing the delivery issues, targeting specific cells, enhancing internalization, and synergizing different therapeutic agents. In addition to serving as drug‐delivery platform, the intrinsic immunomodulatory potential of biomaterials should also be considered when developing biomaterials‐based immunotherapies.^[^ ^25^ ^]^

This review will focus on how biomaterials can assist the targeted modulation of immunological function, especially for the cell‐specific immunomodulation. We begin with a brief discussion of immunological principles regarding to the cancer‐immunity cycle, immunosurveillance, and immune‐resistance. We then dissect the role of biomaterials in targeted modulation of antitumor immunity into three parts: 1) targeted modulation of APCs for enhancing vaccine efficiency; 2) targeted modulation of T cells both ex vivo and in vivo for persistent function or tumor‐homing ability; 3) targeted modulation of specific cells within TME for reversing immunosuppressive TME. By reviewing the current progress in biomaterial‐based cancer immunotherapy and highlighting some state‐of‐art designs, we seek to provide a better understanding of biomaterials’ role in targeted modulation of antitumor immunity as well as to provide guidelines for rationally designing more efficient biomaterials‐based immunotherapies step‐by‐step. Finally, the current challenges and perspectives for potential application and clinical translation of biomaterials‐based immunotherapies will also be discussed.

## 2 The Immunological Principle

### 2.1 Cancer‐Immunity Cycle

To ensure an effective antitumor immune response, a coordinated series of stepwise events, termed as “cancer‐immunity cycle”, must be initiated and allowed to proceed and expand iteratively.^[^ ^26^ ^]^ Tumor‐associated antigens (TAAs) are first generated and released by oncogenesis and captured by APCs, including dendritic cells (DCs), macrophage, neutrophils, and lymphatic endothelial cells, for cellular presenting. Such immunogenic signals might also come from the pro‐inflammatory cytokines and factors released by necrosis, apoptotic tumor cells, or gut microbiota. Afterward, the neoantigens were processed into MHC class I (MHC‐I) and class II (MHC‐II) molecules and presented to effector T cells (naïve CD8^+^ T cells or CD4^+^ T cells), resulting in the activation of immature T cells that reside in the draining lymph nodes (LNs). The activation requires synergistic signaling pathways including: 1) immunological synapsis between antigenic peptide‐bound MHC complex and T cell receptor (TCR); 2) presence of co‐stimulatory factors (ligation of CD80/CD86 on APCs with CD28 on T cells); 3) T cell polarizing driven by cytokines secreted by APCs. Subsequently, the activated tumor‐specific T cells infiltrate into TME and specifically recognize tumor cells through the interaction between TCR and cognate antigen‐bound MHC complex. Finally, the effector T cells kill target cancer cells by inducing apoptotic pathways. Moreover, the death of cancer cells in turn releases additional TAAs, which further strengthens and broadens the revolutions in this cycle. All steps are looped together to construct the overall cancer immunity cycle, which is essential in generating effective antitumor immune responses.

### 2.2 From Immunosurveillance to Immune Evasion

However, one or more steps in the cancer‐immunity cycle might be blocked in cancer patients, leading to the dampened antitumor immune response. This is mainly owing to that tumor can develop multiple mechanisms to evade from immunosurveillance during their progression, a result from immunoediting.^[^ ^27^ ^]^ These mechanisms could directly or indirectly compromise the antitumor immune response. For example, at tumor cell level, reduced immune recognition due to alternation (e.g., loss of strong rejection tumor antigens; loss of MHC‐I or co‐stimulatory molecules; loss of antigen processing function within tumor cells) could promote immune evasion. Moreover, the induction of anti‐apoptotic mechanisms involves persistent activation of pro‐oncogenic transcription factors such as signal transducer and activator of transcription 3 (STAT3) or increased expression of anti‐apoptotic molecules such as B cell lymphoma 2 (Bcl‐2). Other factors that contribute to tumor cell escape include 1) developing an immunosuppressive TME by producing immunosuppressive cytokines such as vesicular endothelial growth factor (VEGF), transforming growth factor *β* (TGF‐*β*), or indoleamine 2,3‐dioxygenase (IDO); 2) recruiting regulatory immune cells that function as the effectors of immunosuppression and; 3) expressing inhibitory co‐stimulatory molecules (such as CTLA‐4, PD1, PD‐L1). Such immune evasion leads to the final outgrowth of tumors that have outstripped immunosurveillance. Therefore, understanding how tumor cell interact with immune system to evade from immunosurveillance or compromise antitumor immunity will provide the opportunities for designing effective cancer immunotherapy strategies.

### 2.3 Resistance to Cancer Immunotherapy

Benefit from these understanding, various immunotherapies designed to circumvent immune evasion and to resume the cancer‐immunity cycle are now being developed.^[^ ^28^ ^]^ For example, cancer vaccine is designed to promote APCs maturation and enhance antigen presentation from APCs to T cells, leading to activation of antitumor immune response. ICB therapy is designed to block the inhibitory molecules to restore the CTL activity against tumor cells. CAR‐T cells therapy is developed by genetically engineering T cells to express tumor antigen‐specific receptor and expanding ex vivo and then injected back to patients to kill tumor cells. Although cancer immunotherapies can induce considerable and durable antitumor immune responses, clinical benefits have only been observed in a small population of patients, most patients have limited or even no response.^[^ ^29^ ^]^ The heterogeneous responses have been observed among patients and among different tumors, which is partly owing to that tumor cells has the ability to resist the immunotherapies. Clinically, current immune resistances can be divided into two types: 1) primary or adaptive resistance which prevents a patient from ever responding to an immunotherapy; 2) acquired resistance which facilitates relapse after an initial response.^[^ ^29^ ^]^ Although resistance to immunotherapies may manifest at different times, in many cases, the mechanisms seem to broadly overlap with those involved in natural progression of tumors undergoing immunoediting to escape immunosurveillance.^[^ ^30^ ^]^ Accumulating evidence from patients receiving various forms of cancer immunotherapies revealed that cancer immunoediting reoccurs either in part or in its entirety during therapy.^[^ ^31^ ^]^ Given the existence of immune resistances, strategies to maintain antitumor immune response in an overwhelming level while also to overcome immune resistance simultaneously is critical for achieving clinical benefits. Therefore, broadening the clinical implementation of immunotherapies and predicting their therapeutic efficacy require a better understanding of underlying mechanisms of resistance to immunotherapies.

## 3 Biomaterial‐Assisted Targeted Modulation

**Figure 1:**
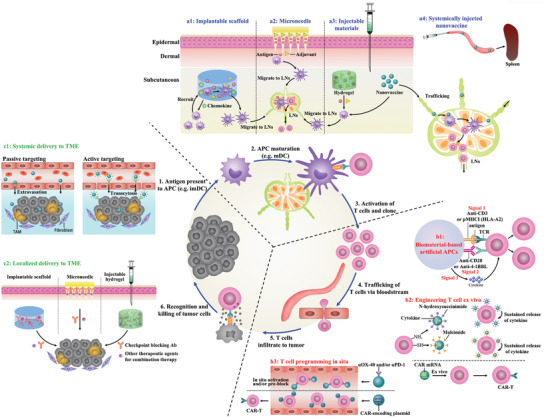
Biomaterials‐assisted cell‐specific modulation along the cancer‐immune cycle. The cell‐specific modulation can be generally divided into three parts. Part A: Delivering cancer vaccine to APCs resident in lymphoid organs to prime antitumor immune response via different administration routes, such as targeting skin‐resident DCs, LN‐resident DCs, and spleen‐resident DCs. Part B: Targeting modulation of effector T cell both ex vivo and in vivo, such as ex vivo expansion of T cells using artificial APCs (aAPCs), conjugating cytokine‐containing NPs onto T cell surface ex vivo, and in situ T cell programming. Part C: Targeting modulation of tumor cell or immune cell within TME based on systemic delivery or localized delivery technologies.

In addition to intrinsic immune resistances, the limited delivery efficiency and specificity of immunotherapies also contribute to the modest therapeutic outcome. Increasing the administration dose may enhance the therapeutic effect to a certain extent, while patients may be subjected to even higher risk of irAEs or off‐targeting side effects. Therefore, great control over how these immunological cues delivered, presented, and processed specifically will be greatly important for maximizing the therapeutic potential while minimizing the irAEs. Encouragingly, the use of biomaterials for targeting delivery of immunological cues has emerged as promising and widely accepted strategy. By rationally designing, biomaterials with unique properties and functionalities can home to specific cells within the cancer‐immune cycle respectively or synergistically, thus enabling targeted modulation of antitumor immunity (**Figure** **1**). This biomaterial‐assisted cell‐specific modulation can be generally divided into three parts based on the function of different immunotherapies. 1) Biomaterials‐based cancer vaccine for APCs‐specific modulation: in this part, we will review how biomaterials can deliver vaccine components specifically to APCs resided in different lymphoid organs (e.g., LNs, spleen and skin) to prime antitumor immunity as well as their design rationale. 2) Biomaterials‐assisted T cell engineering: in this part, we mainly focus on how biomaterials can act as either immunomodulatory tool or delivery platform to engineer effector T cells both ex vivo and in vivo for persistent T cells functionality or in situ endowing of tumor‐homing ability. 3) Biomaterial‐assisted reversion of immunosuppressive TME: as a major milieu where the immunosuppressive signals occur, we mainly focus on how biomaterials can selectively deliver immunological cues to TME as well as to distinct cell populations within TME to circumvent immunosuppressive mechanisms by systemic and localized administration, and eventually reverse the immunosuppressive TME to restore antitumor immunity. By dissecting the role of biomaterials in mediating targeted modulation of anti‐tumor immunity step‐by‐step, we seek to provide better understanding on the design rationales for biomaterials‐based immunotherapies as well as the current advances.

## 4 Biomaterial‐Based Targeting Modulation of APCs

APCs play a pivotal role in the response to vaccines, bringing about either immunity or tolerance.^[^ ^32^ ^]^ As one of the professional APCs, DCs are particularly important for initiating effective adaptive antitumor immune response because they govern the activation of CD4^+^ and CD8^+^ T cells.^[^ ^33^ ^]^ Thus, targeting modulation of DCs, such as delivering antigen and/or adjuvant to DCs, enhancing antigen uptake, promoting DCs maturation and antigen presentation, may hold great promise to elicit strong antitumor immune response. So far, various biomaterials can be rationally tailored to specifically target different subsets of DCs in vivo in distinct pathways.^[^ ^34^ ^]^ For example, interstitially injected nanovaccine enables targeting delivery to LNs‐resident DCs, implantable scaffold, MN patch or injectable hydrogel enables localized delivery of vaccine to skin‐resident DCs, as well as systemically injected nanovaccine enables targeting delivery to spleen‐resident DCs. To deliver the vaccine components specifically to DCs, the ability to deliver vaccine components to specific lymphoid organs is a prerequisite. The rationales for designing biomaterials that can circumvent rapid clearance, prolong circulation time, and improve pharmacokinetic profiles have been well reviewed previously.^[^ ^35^ ^]^ Instead, we mainly focus on design rationale for lymphoid organs‐targeting delivery of cancer vaccine based on the administration route.

### 4.1 Nanovaccine‐Based Passively Targeting Delivery to LN‐Resident APCs

LNs, the secondary lymphoid organs, are situated in lymphatic drainage pathways and contain a large, various populations of immune cells, including T cells, B cells, and APCs (mainly referred to DCs and macrophages).^[^ ^36^ ^]^ Since the cellular and humoral immunity in cancer surveillance is mainly orchestrated by these immune cells within LNs, targeting LNs is a promising strategy for controlling vaccine efficacy in both prophylactic and therapeutic setting. Intranodal injections have shown great promise in directly and efficiently delivering vaccines to LNs, while this administration pathway requires an invasive surgical operation or ultrasound‐directed guidance.^[^ ^37^ ^]^ Instead, interstitial injections (e.g., subcutaneous (s.c.), intradermal (i.d.), and intramuscular (i.m.)) are more accessible administration routes and are widely used for vaccination.^[^ ^38^ ^]^ However, the effectiveness of conventional cancer vaccine is limited by the pre‐existing biological barriers that prevent interstitially administrated vaccine from reaching LNs. To troubleshoot this issue, the use of NPs to deliver vaccine components specifically to LNs has emerging as an attractive alternative. Nanoparticulate vaccines or so‐called nanovaccines, generally via interstitial administration, can be designed to target LNs via passive interstitial drainage.^[^ ^39^ ^]^

Accumulating evidence suggested that the physicochemical properties of nanovaccine, including size, charge, shape, surface properties, can affect LNs‐targeting efficiency.^[^ ^40^ ^]^ Among these, NPs’ size has been shown to play a critical role in determining the LN‐targeting trafficking and retention. It has been demonstrated that a small‐size NPs with an average size of sub‐100 nm can traverse the interstitial space by diffusion and convection transport through water‐channels in the dense extracellular matrix (ECM), cross the weak cell–cell junctions (100 nm in size) between neighboring lymphatic endothelial cells or discontinuous basement membrane, and subsequently drain into the LNs within hours post‐injection.^[^ ^41^ ^]^ Reddy et al., reported that 25‐nm pluronic copolymer‐coated NPs efficiently homed to LNs through interstitial drainage and retained for at least 120 h after i.d. injection. In contrast, the efficiency of 100‐nm NPs accumulated at LNs demonstrated only ≈10% as efficient. As a consequence, the 25‐nm NPs were able to elicit stronger humoral immune response than 100‐nm after loading with the model antigen ovalbumin (OVA).^[^ ^42^ ^]^ More specifically, it is now considered that sub‐50 nm NPs may possess stronger migratory capacity and tend to target LN more efficiently.^[^ ^43^ ^]^ For example, Howard et al. developed three sets of poly(lactic‐*co*‐glycolic acid)‐*b*‐poly(ethylene‐glycol) (PLGA‐*b*‐PEG) with number average diameter of 20, 40, and 100 nm and compared their LN‐targeting efficiency. The result showed 20 nm NPs drain rapidly across proximal and distal LNs following subcutaneous inoculation in mice and is retained in LNs more effectively than 40 nm NPs, while 100 nm NPs rarely drain to LNs. Besides, 20 nm NPs showed the highest degree of penetration around the paracortex region and had enhanced access to DCs in the LNs.^[^ ^44^ ^]^ However, in another study, Zhang and Chan et al., reported a contradictory result that gold NP‐based nanovaccine with a size of 50–100 nm are more efficient in inducing humoral immune response than 5–15 nm nanovaccine. They discovered that the follicular DCs networks determined the intra‐LN distribution fate of nanovaccine. Smaller nanovaccine (5–15 nm) were likely to be cleared within 48 h while larger nanovaccine (50–100 nm) tend to retain at follicle for over 5 weeks. They also demonstrated that follicular DCs are prone to internalize smaller nanovaccine while align larger nanovaccine on their surface. Therefore, 50–100 nm nanovaccine could deliver 175‐fold higher antigen to follicular DCs than 2–15 nm nanovaccine, leading to much enhanced humoral immune response.^[^ ^45^ ^]^ The contradictory reports may be attributed to the different composition, rigidity as well as morphology. As described above, if the size of nanovaccine is too small, they may have the chance to cross the vascular capillaries and preferentially enter blood vessels since the junction between neighboring vascular endothelial cells is less than 10 nm.^[^ ^46^ ^]^ One study based on dendrimers reported that particles with a size smaller than 6 nm usually drain into the blood, while particles with size larger than 9 nm preferentially drain to the lymphatic system.^[^ ^47^ ^]^ It should be noted that nanovaccine with a size over 100 nm tend to be excluded from directly entering lymphatic vessels via passive diffusion and is often taken up by tissue‐resident DCs at the injection site, which carry internalized nanovaccine to LNs through migration.^[^ ^43^, ^48^ ^]^ However, this route seems to be less LN‐targeting efficiency because it often takes ≈24 h for them to arrive in LNs.^[^ ^49^ ^]^ Therefore, the ideal size range of interstitially administrated NPs for efficient LN‐targeting through lymphatic drainage should be 10–100 nm, while the best size range for nanovaccine with specific composition requires more investigation.

**Figure 2:**
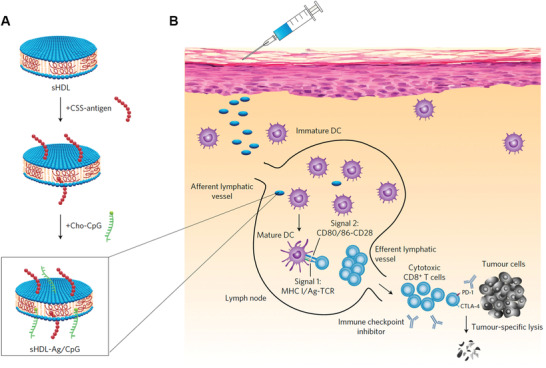
Nanovaccine passively target LNs‐resident APCs. A) Schematic illustration of the preparation and composition of sHDL nanodisk. B) Schematic illustration of mechanism of sHDL nanodisk for targeting delivery to LNs and induce tumor‐specific T cells activation. Reproduced with permission.^[^ ^50^ ^]^ Copyright 2017, Nature Publishing Group.

Based on the design features, numerous nanovaccines with a size between 10–100 nm have been developed for LNs‐targeting delivery and showed promising antitumor efficiency. Upon entering LNs, nanovaccines are often easily recognized, captured, and ingested by LNs‐resident APCs due to their powerful phagocytic ability, presenting a passive behavior. These APCs can then process and present specific phenotype to naïve T cells resident in same LNs, leading to the activation and expansion of phenotype‐specific T cells. For example, Kuai and Moon et al. proposed a synthetic high‐density lipoprotein (sHDL)‐based nanodisk vaccine co‐loaded with neoantigen and adjuvant for personalized cancer vaccine (**Figure** **2**A,B).^[^ ^50^ ^]^ The neoantigen peptides, a single‐epitope mutation within Adpgk protein in MC‐38 colon carcinoma, were modified with cysteine–serine–serine (CSS) linker, which can conjugate onto sHDL nanodiscs. Meanwhile, the adjuvant cytosine–phosphodiester–guanine (CpG) conjugated with cholesterol (Cho‐CpG) was further inserted into sHDL nanodisks. The nanodisk vaccine with an average diameter of 10.5 nm was showed to preferentially trafficking to LNs and to be captured by LNs‐resident APCs to activate tumor‐specific T cells. Therefore, strong anti‐tumor immune response with 47‐fold greater frequencies of neoantigen‐specific CTLs was activated to inhibit tumor growth. Moreover, this nanodisk could be customized with other antigenic peptide to develop more specific and personalized cancer vaccine. In a recent study conducted by same group, they developed aldehyde dehydrogenase (ALDH) and CpG co‐loaded nanodisk vaccine with a size around 10 nm for treatment of ALDH^high^ cancer stem cells (CSCs).^[^ ^51^ ^]^ In vivo studies further confirmed the excellent antitumor efficiency in both D5 melanoma and 4T1 breast cancer model after combined vaccination of the nanodisk vaccine and anti‐PD‐L1 antibody (*α*PD‐L1). These studies further supported that nanovaccine with a small size can efficiently improve the accumulation of antigen in LNs. In addition to size, the surface charge may also play an important role in determining the uptake efficiency by APCs. For example, Lynn et al. developed a self‐assembling nanovaccine platform (SNP‐7/8a) based on charge‐group modified (CM) long peptide conjugated with toll‐like receptor (TLR)‐7/8 agonist (CM‐LP oligo‐7/8a, termed as CM conjugates).^[^ ^52^ ^]^ Upon resuspension in aqueous solution, the hydrophobic block of CM conjugates undergoes multimerization while the charge‐modifying group provide a countervailing force, leading to the self‐assembly of NP micelles with uniform size (≈20 nm) regardless of the peptide antigen composition. However, without CM, the conjugates may form large microparticles and aggregates (MP‐7/8a) (Figure 2C,D). They found that CM conjugates with a positive charge can lead to smaller size than that with a negative charge. The fluorescence intensity of draining LNs at immunization site after s.c. injection with only AF647‐labeled LP SNP‐7/8a or LP‐SNP‐7/8a&polyICLC (adjuvant) was much higher than that injected with native LP&PP‐7/8a or native LP&polyICLC. Further quantitative analysis showed that immunization with LP‐SNP‐7/8a&polyICLC led to highest percentage of AF647^+^CD11c^+^ DCs, suggesting LP‐SNP‐7/8a could efficiently deliver to LNs and LNs‐resident DCs. More importantly, LP‐SNP‐7/8a alone could stimulate much higher CD8^+^ T cells activation than native LP&PP‐7/8a or native LP&polyICLC treatment 7 days post‐immunization. They also reported that LP‐SNP‐7/8a could induce equivalent T cell response regardless of s.c. administration or intravenous (i.v.) administration. Immunization with neoantigen‐conjugated SNP‐7/8a by either s.c. administration or i.v. administration could significantly suppress tumor growth compared to control peptide‐conjugated SNP‐7/8a. This approach offers a strategy to develop nanovaccine with positive charge by conjugating diverse positively CM peptide neoantigens with TLR‐7/8a adjuvant, which increased uptake by and activation of DCs that further induce T cell activation. This study indicated that nanovaccine with a positive surface charge is generally internalized by DCs more efficiently than those with a natural or negative charge. Moreover, other factors such as geometry, kinetic, surface modification, hydrophobicity may also affect the efficiency of nanovaccines.^[^ ^43b^ ^]^

### 4.2 Nanovaccine‐Based Active Targeting Delivery to LN‐Resident APCs

Although size‐dependent passive targeting delivery to APCs represents a promising strategy for priming antigen‐specific CLT, there are still several challenges in efficiently delivering to DCs. For example, nanovaccies are often functionalized with hydrophilic PEG layer or anionic surface coating to improve biodistribution to LNs, their uptake by APCs can thus be hindered by steric hindrance or electrostatic repulsion.^[^ ^53^ ^]^ Additionally, mature DCs (mDCs) are reported to abundantly co‐exist with immature DCs (imDCs) and exhibit stronger antigen presenting ability, while their phagocytic ability dramatically decrease during maturation.^[^ ^43^, ^54^ ^]^ Therefore, active targeting to LN‐resident APCs has also been explored based on size‐dependent targeting strategy by grafting specific ligand onto the surface of nanovaccines.^[^ ^55^ ^]^ Upon draining to LNs, these ligands‐modified nanovaccines specifically bind to the surface receptors expressed by APCs, leading to enhanced uptake by APCs. To date, numerous APC‐specific receptors, including Fc receptors (FcRs), C‐type lectin receptors (CLRs), CD40, CD11c, and scavenger receptor class B1 (SR‐B1), have been well identified and harnessed for targeted delivery of nanovaccines, as summary in **Table** **1**. Nanovaccines can be targeted to FcRs by full antibodies or Fc fragments, with most studies targeting Fc*γ* receptor.^[^ ^56^ ^]^ However, the immunological outcome of these strategies depends on the balance between activating and inhibitory signals induced by triggering of the various Fc*γ* receptors expressed by DCs. CLRs represent a family of receptors that bind to specific carbohydrate residues in a calcium‐dependent manner via their carbohydrate‐recognition domain, such as mannose, DEC‐205, dendritic cell‐specific ICAM3‐grabbing nonintegrin (DC‐SIGN) receptors, DC‐associated C‐type lectin‐1 (Dectin‐1), lectin‐like oxidized low‐density lipoprotein receptor 1 (LOX‐1), and C‐type lectin domain containing 9A (Clec9A).^[^ ^57^ ^]^ For example, both inorganic and polymeric NPs have shown enhanced binding and uptake by DCs when coupled with antibodies against CD40, CD11c, or DEC‐205 receptors.^[^ ^58^ ^]^

**Table 1:** Summary of nanovaccives modified with different ligands for active DCs targeting

Receptor	Ligand	Carrier	Therapeutic cargo	Tumor type	Administration route	Ref.
DEC‐205	*α*DEC‐205	Liposome or PMV	OVA/peptide	B16 melanoma metastasis	i.v.	[65]
	*α*DEC‐205	Polymeric NPs	OVA	EL4 lymphma	s.c.	[66]
	*α*DEC‐205	PLGA NPs	OVA&polyI:C&R848	N/A	N/A	[67]
	*α*DEC‐205	PLGA NPs	OVA	N/A	i.p.	[68]
	*α*DEC‐205	PLGA NPs	OVA	B16 melanoma	s.c.	[69]
	*α*DEC‐205	Liposome	OVA&EUPS	N/A	s.c.	[70]
DC‐SIGN	glycan lewis	liposome	gp100/MART‐1	N/A	i.t.	[71]
	triMN	lipid‐polymer‐RNA lipopolyplex	E7/OVA/MART‐1	TC‐1 tumor/ E.G7 lymphoma/ B16F10 melanoma	s.c.	[72]
	aptamer	CMV	CpG	CT26 colon	i.t.	[64]
CD40	*α*CD40	PLGA NPs	OVA	B16 melanoma	s.c.	[69]
	Agonistic *α*CD40	*γ*‐PGA NPs	Agonistic *α*CD40	Bladder cancer	s.c.	[73]
	*α*CD40	PLGA NPs	OVA	B16 melanoma	s.c.	[74]
CD11c	*α*DEC‐11	Liposome or PMV	OVA/peptide	B16 melanoma metastasis	i.v.	[65]
	*α*CD11c	PLGA NPs	OVA	B16 melanoma	s.c.	[69]
	*α*CD11c	Lipos‐AuNCs	TRP2 peptide	B16 melanoma	s.c.	[75]
MR	mannose	chitosan NPs	tumor cell lysate	B16 melanoma	s.c.	[76]
	mannose	alginate NPs	OVA	E.G7 lymphoma	s.c.	[77]
	mannose	lipid‐polymer NPs	OVA&IMQ	E.G7 lymphoma	s.c.	[63]
	mannose	polymersome	OVA/MPLA/IMQ	E.G7 lymphoma	s.c.	[78]
	mannose	calcium NPs	pOVA	E.G7 lymphoma	s.c.	[79]
SR‐B1	*α*‐peptide	peptide‐antigen NPs	OVA peptide/gp100 peptide	E.G7 lymphoma	s.c.	[80]

**Figure 3:**
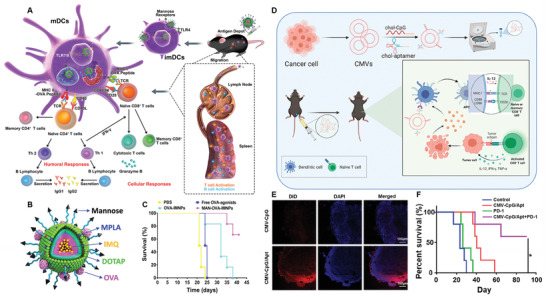
Ligand‐modified nanovaccine for active targeting delivery to LNs‐resident APCs. A) Illustration of the targeted co‐delivery of antigen and adjuvant by MAN‐OVA‐IMNPs to LNs‐resident DCs and subsequent activation of antigen‐specific T cells. B) Schematic illustration of the structure of MAN‐OVA‐IMNPs. C) E.G7‐OVA‐bearing mice survival after treatment with MAN‐OVA‐IMNPs and control formulations. Reproduced with permission.^[^ ^63^ ^]^ Copyright 2019, American Chemistry Society. D) Schematic illustration of preparation of CMVs‐CpG/Apt and their function to induce antitumor immunity. E) Confocal images of LNs slices treated with CMVs‐CpG/Apt and CMVs‐CpG. F) CT26 tumor‐bearing mice survival after treatment with CMVs‐CpG/Apt+*α*PD1 and control formulations. Reproduced with permission.^[^ ^64^ ^]^ Copyright 2021, American Chemistry Society.

Among these, mannose receptor (CD206) is a type I membrane transmembrane protein that is highly expressed on APCs (e.g., DCs and macrophages),^[^ ^59^ ^]^ lymphatic epithelium,^[^ ^60^ ^]^ and also overexpressed on the surface of many malignant tumor cells.^[^ ^61^ ^]^ Given its crucial role in both the innate and adaptive immunity by mediating uptake of soluble protein antigens into APCs, mannose receptor is now being widely exploited as a promising target for targeted modulation of DCs or macrophages to induce antitumor immunity.^[^ ^62^ ^]^ To enhance DC‐targeting and vaccine efficiency, Zhang et al. constructed a novel versatile and mannose‐modified lipid‐polymer hybrid nanovaccine (MAN‐OVA‐IMNPs) for a targeting codelivery of antigen and dual TLR agonists.^[^ ^63^ ^]^ MAN‐OVA‐IMNPs consists of four distinct components: 1) a hydrophobic inner core self‐assembled block copolymer for encapsulation of hydrophobic TLR7/8 agonist imiquimod (IMQ); 2) a lipid layer for the incorporation of TLR4 agonist monophosphoryl lipid A (MPLA); 3) a cationic DOTAP lipid for the electrostatic adsorption of the anionic OVA antigen, and 4) a MAN‐targeting moiety functionalized on the outer lipid layer with PEG layer. MAN‐OVA‐IMNPs could specifically deliver to LNs after s.c. injection and be efficiently internalized by imDCs by recognizing mannose receptor (MR) on their surfaces. After internalization, the OVA antigen could be released from NPs and escaped from the endosome/lysosome due to the “sponge effect” of DOTAP, leading to an antigen peptide presented by both MHC‐I and MHC‐II pathways. In the meanwhile, MPLA was delivered to extracellular TLR4 and IMQ was delivered to intracellular TLR7/8 simultaneously, resulting in a synergistic DCs activation through both the myeloid differentiation primary‐response gene 88 (MyD88) and the TIR‐domain‐containing adaptor protein inducing INF‐*β* (TRIF) pathways (**Figure** **3**A–C). Both in vitro and in vivo studies demonstrated that MAN‐decoration could significantly improve DC uptake compared to unmodified one and free agonists. Vaccination with MAN‐OVA‐IMNPs induced the highest frequency of 79.41% of antigen‐specific CD8^+^ T cells and memory T cells compared with control groups, suggesting the great potential of MAN‐OVA‐IMNPs in inducing antitumor immunity. Most importantly, pre‐immunized with MAN‐OVA‐IMNPs significantly delay tumor growth and prolong the survival of mice after E.G7‐OVA cells challenging compared to control group, suggesting the excellent prophylactic effect of MAN‐OVA‐IMNPs. Moreover, combination treatment of MAN‐OVA‐IMNPs with the anti‐PD1 antibody (*α*PD1) further improved the therapeutic effect.

In another study, Liu et al. designed a targeted nanovaccine by modifying tumor cell‐derived cell membrane vesicle (CMVs) with CpG and aptamer that specifically target DC‐SIGN on DCs (Figure 3D–F). Instead of encapsulating immune adjuvant inside NPs, the CpG was coated onto the CMVs. Following i.d. administration, such CpG and aptamer‐modified CMVs (CMVs‐CpG/Apt) could traffic to LNs and target DC‐SIGN^+^ DCs to improve the uptake of CpG, leading to DCs maturation and subsequent T cells activation. In vitro study showed that CMVs‐CpG/Apt could trigger more DCs maturation compared to CMVs‐CpG without aptamer modification and other formulations. In vivo distribution exhibited significantly better LNs‐targeting efficiency and DCs maturation in mice treated with CMVs‐CpG/Apt than CMVs‐CpG, indicating that aptamer could mediate efficient DCs targeting. Moreover, treatment with CMVs‐CpG/Apt greatly suppressed the B16‐OVA tumor cells growth compared to other treatments, leading to much improved survival. Combination with *α*PD1 demonstrated a further improved treatment benefit and induced remarkable memory antitumor immunity against tumor rechallenging. All the studies validate the effectiveness of ligand, aptamer or antibody can enable effective active targeting delivery of nanovaccine to DCs and improve DCs performance.

Another strategy to efficiently target APCs was described as the “albumin hitchhiking” approach. Inspired by clinical finding that injected dyes that bind avidly to endogenous albumin were then efficiently transported to LNs, where they were filtered by resident APCs.^[^ ^81^ ^]^ Liu et al. developed an amphiphilic macromolecule (amph‐vaccines) to specifically deliver antigen or adjuvant to LNs.^[^ ^82^ ^]^ The amphiphile comprised an antigen or adjuvant cargo linked to a lipophilic albumin‐binding tail, with a hydrophilic PEG polymer chain to improve solubility. After s.c. administration into mice, these amph‐vaccines were found to bind the endogenous albumin and transport to LNs, targeting DCs and macrophage. In vivo trafficking studies demonstrated that amph‐vaccines accumulated in LNs eightfold higher than soluble CpG 24 h post‐injection and retained in LNs for 3 days. More importantly, vaccination with the amphiphilic peptide antigens and CpG adjuvant led to significant increase in antigen‐specific CTL priming and antitumor therapeutic efficacy while great decrease in systemic toxicity compared to unmodified peptide and CpG immunizations. It was shown that amph‐vaccines induced sustained regression of large TC‐1 tumors that were only modestly affected by soluble vaccines and delayed the growth of B16F10 melanoma, in which a traditional soluble vaccine had no effect. These results validate the effectiveness of “albumin hitchhiking” in targeting delivery antigen or adjuvant to LNs‐resident APCs.

### 4.3 Targeting Delivery to TDLNs‐Resident DCs

Tumor‐draining lymph nodes (TDLNs) lie immediately downstream of tumors and are the site to which tumor antigens first drain and tumor‐derived DCs migrate.^[^ ^41^, ^83^ ^]^ Increasing evidence suggests that the TDLN, although in small size, plays a determinant role in setting the course of the subsequent immune response to tumor. Unfortunately, the local microenvironment of TDLNs undergoes active and ongoing alternation from a site favoring immune activation into a site favoring immune suppression and tolerance. In particular, the metastatic TDLNs, referred to LNs invaded by metastatic cancer cells, is involved in supporting tumor progression and metastasis.^[^ ^84^ ^]^ These alternations driven by upstream tumor‐derived factors are often characterized by lymphangiogenesis, tolerogenic DCs, recruitment or expansion of immunosuppressive cells and upregulation of chemokines and cytokines, making TDLNs as immune privilege site and leading to tolerogenic induction.^[^ ^83^, ^85^ ^]^ The tolerogenic mechanism are so potent in the TDLNs that any new antigens, regardless of experimental xenoantigen or spontaneous neoantigen arising in the tumor, may induce tolerance to itself.^[^ ^83^ ^]^ Since TDLNs are already bathed in abundant TAAs, delivering adjuvants alone might be sufficient to induce an anti‐tumor immune response. For instance, Thomas et al. developed 30 nm polymeric NPs loaded with CpG (CpG‐NP) or paclitaxel (PXL‐NP) that effectively target DCs (CD11c^+^) within TDLNs and induced DC maturation.^[^ ^86^ ^]^ After administration daily in the limb ipsilateral (i.l.), CpG‐NP induced DC maturation within the TDLNs and reshaped the CD4^+^ T cell distribution within the tumor toward a Th1 (CXCR3^+^) phenotype. This treatment also led to an increase in the frequency of antigen‐specific CD8^+^ T cells within the tumor and thus a slower tumor growth rate compared to contralateral (c.l.) delivery of CpG‐NP (non‐TDLN targeting) or i.l. delivery of free CpG. Additionally, treatment with PXL‐NP treatment reduced the frequency of regulatory T (FoxP3^+^ CD4^+^) cells in the TDLNs. Together, these data implicated the feasibility of targeting delivery of adjuvant to TDLN for solid tumors therapy compared to targeting other kinds of LNs.

### 4.4 Nanovaccine‐Based Targeting Delivery to Spleen‐Resident APCs

Spleen is the largest secondary lymphoid organ which evolves concurrently with adaptive immunity based on rearranging immunoglobulin (Ig) and TCR.^[^ ^87^ ^]^ Based on its histological structure and function, spleen can be divided into two discrete regions: the red pulp (RP) and the white pulp (WP).^[^ ^88^ ^]^ The RP is mainly responsible for filtration of blood, including removal of abnormal erythrocytes and pathogen capture. In contrast, the WP of spleen is the primary immunologic region where stores a wide range of immune cells, including T cells, B cells, and APCs under resting conditions, which plays a key role in initiating adaptive immunity.^[^ ^87b^ ^]^ Unlike LNs, the spleen lacks afferent lymphatic vessels, leading to that all cells and antigens enter spleen via the blood. Therefore, systemic delivery of immunomodulatory molecules to spleen to elicit effective antitumor immune response may represent another promising strategy. It has been reported that NPs with a hydrodynamic size over 200 nm are more likely to activate the human complement system and thus be phagocytosed by the live‐ and spleen‐resident macrophage and DCs, resulting in preferential accumulation in liver and spleen.^[^ ^89^ ^]^ In addition to be captured by spleen‐resident macrophage and DCs, other studies reported that splenic filtration also contributes to retention of NPs > 200 nm due to the 200–500 nm size range of interendothelial cell slits.^[^ ^90^ ^]^ Meanwhile, the composition and surface charge also play an important role in controlling the homing to spleen. Positively charged NPs are more prone to sequestrated by liver‐ and spleen‐resident macrophage compared to neutral and negatively charged NPs,^[^ ^91^ ^]^ while it may depend on the composition. For example, Kranz et al. constructed RNA‐lipoplexes (RNA‐LPX) nanovaccines for systemic delivery of message RNA (mRNA) to spleen to induce antitumor immunity.^[^ ^92^ ^]^ By optimizing the ratio of lipid:RNA, the composed RNA‐LPX with size of 200–320 nm were demonstrated mainly locating in spleen, indicating both size and surface charge are critical factors for spleen‐targeting delivery of NPs, without the need of targeting ligands. The excellent spleen‐targeting delivery promoted the authors to explore the antitumor potential of RNA‐LPX. They found that the RNA‐LPX was mainly internalized by spleen‐resided macrophages and DCs, while DCs showed a higher translation efficiency of RNA that encoding TAAs. As a result, RNA‐LPX could induce strong effector and memory T cell response, and thus suppress the tumor progression in an interferon *α* (IFN‐*α*)‐dependent manner post‐injection systemically. Moreover, the therapeutic effect was further moving onto a phase I clinical trials with three advanced melanoma patients (NCT02410733). The patients were intravenously administrated with a very low initial dose of RNA‐LPX vaccine that encoding four tumor antigens (NY‐ESO‐1, MAGE‐A3, tyrosinase and TPTE), followed by four weekly treatments with moderately higher doses. Encouragingly, all patients had dose‐dependent early release of IFN‐*α* and C‐X‐C motif ligand 10 (CXCL‐10) peaking at 6 h post‐injection and were well‐tolerated with transient flu‐like symptoms. Taking together, systemically injected NPs with a size range between 200–500 nm are more likely to deliver preferentially to spleen, which can be leveraged to develop nanovaccines.

As aforementioned above, senescent and damaged erythrocytes are physiologically eliminated by scavenger cells, such as macrophage and DCs in the spleen. It can be hypothesized that red blood cells (RBCs) possess intrinsic spleen tropism and could be leveraged as an alternative platform to deliver immunomodulatory molecules to spleen. In another study, Han et al. developed nanosized RBC‐tumor membrane vesicles or nanoerythrosomes by fusing TAAs‐carried cancer cell membrane and RBC membrane.^[^ ^93^ ^]^ In vivo studies demonstrated that the fused “Nano‐Ag@erythrosome” was rapidly cleared from blood into spleen post i.v. injection, verifying that nanoerythrosome could be an effective spleen‐targeting delivery platform. Besides, the TAAs carried by nano‐Ag@erythrosomes could be efficiently captured by MHC‐II^+^ splenic APCs and thus induced their maturation, which further led to activation of splenic natural killer (NK) cells, B cells, CD4^+^ T cells and CD8^+^ T cells. Given to the enhanced antitumor immunity, they further validated that the combination of nano‐Ag@erythrosome and *α*PD‐L1 led to significant tumor regression in both B16F10‐luc and 4T1 tumor‐bearing mouse model. The excellent spleen‐targeting delivery efficiency and therapeutic outcome make RBC membrane a good candidate as personalized vaccination and provide a thinking to design spleen‐targeted vaccines.

### 4.5 Biomaterial‐Based Targeting Delivery to Skin‐Resident APCs

The skin, as a primary interface between the body and environment, provides the first line of defense against a broad array of microbial pathogens, physical and chemical insults.^[^ ^94^ ^]^ In addition to its well‐characterized mechanical barrier function which physically restricts water loss and prevents the entry of potentially harmful environmental substances and microorganisms, the skin is also an active protective barrier serving as the immune surveillance system.^[^ ^94a^ ^]^ The skin is composed of the epidermis, attached to a basement membrane, underlain by the dermis and a subcutaneous fatty region. Both the epidermis and dermis are populated by a variety of cell types that together form an orchestrated defense against invading microorganisms.^[^ ^95^ ^]^ In the epidermis, Langerhans cells (LCs) are the major skin‐resident APCs and CD8^+^ T cells can be found in the stratum basale and stratum spinosum. The underlying dermis is anatomically more complicated, with various cell diversities. It contains many specialized immune cells, including DCs, macrophages, CD4^+^ T helper (T_H_) cells, and innate lymphoid cells (ILCs). It should be noted that there are species‐specific differences in the immune cells populations that colonized in the epidermis and dermis.^[^ ^96^ ^]^ Moreover, these skin‐resident immune cells also play a fundamental role in the surveillance of subclinical epidermis‐derived skin cancers such as melanoma by maintaining cancer‐immune equilibrium.^[^ ^97^ ^]^ Therefore, delivering vaccines to specific skin‐resident APCs may prime strong local immune response against peripheral cancer. However, it remains challenging to target skin‐resident immune cells specifically while avoiding off‐target effect due to the physical barrier.^[^ ^98^ ^]^ To troubleshoot this, biodegradable MN patch,^[^ ^19^ ^]^ injectable hydrogel^[^ ^18^ ^]^ and implantable scaffold^[^ ^20^ ^]^ that enable intradermal or transdermal delivery of immunological cues to skin‐resident APCs have been regard as reliable and effective vaccination strategy.

#### 4.5.1 MN‐Based Modulation of Intradermal and Subcutaneous APCs

**Figure 4:**
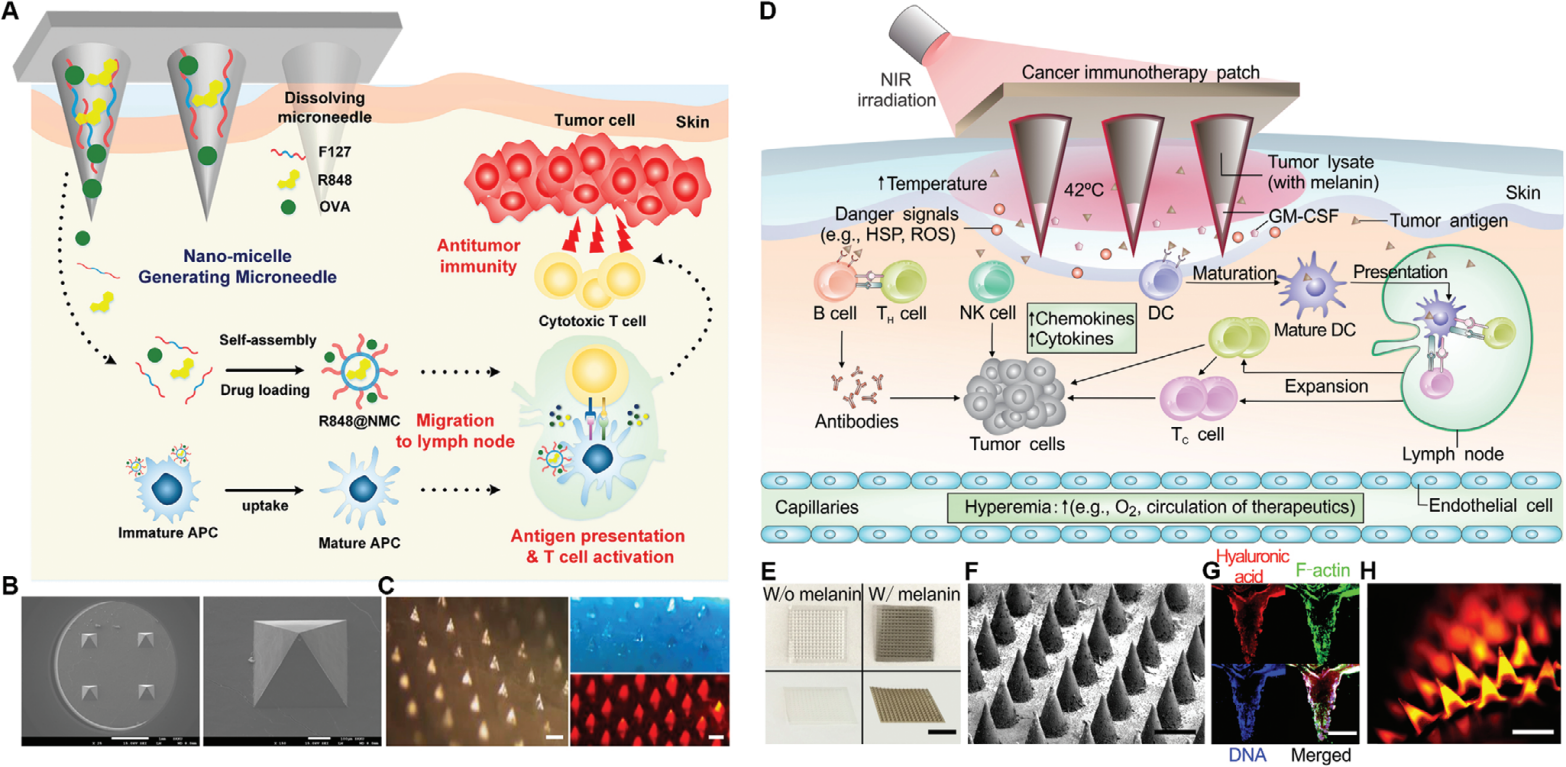
MN‐based vaccine for modulation of intradermal and subcutaneous DCs. A) Schematic illustration of the mechanism of OVA/R848 MN. Upon dissolving post‐insertion, the in situ formation of NMCs assisted the delivery of hydrophobic R848 and OVA to LNs. B) Scanning electron microscope (SEM) and C) fluorescence stereomicroscope images of DiD‐loaded MNs. Reproduced with permission.^[^ ^100^ ^]^ Copyright 2018, American Chemical Society. D) Schematic illustration of mechanism of this NIR‐responsive transdermal MN vaccine. E) Photograph of representative MN patches without (W/o) and with (W/) melanin (scale bar, 4 mm). F) SEM image of the MN patch (scale bar, 400 µm). G) Fluorescence cross‐sectional images of a representative MN. H) Fluorescence imaging of a representative MN patch that contained the Alexa Fluor 488 phalloidin‐labeled tumor lysate and rhodamine B‐labeled hyaluronic acid (scale bar, 400 µm). Reproduced with permission.^[^ ^101^ ^]^ Copyright 2017, American Association for the Advancement of Science.

In an early study, Zaric et al. developed a polymeric MN encapsulated with antigen‐loaded PLGA NPs for i.d. delivery to skin‐resident DCs.^[^ ^99^ ^]^ Following insertion, the polymeric MN patch could be dissolved gradually to release the antigen‐loaded PLGA NPs, which could be further captured and uptake by intradermal DCs. These antigen‐carried DCs then migrated to subcutaneous LNs where they could stimulate antigen‐specific T cells activation and expansion. In vivo studies proven that this nanovaccine‐encapsulated MN induced robust cellular immune response in mice, leading to an effective protection in the mice against the challenging of antigen‐expressing B16 melanoma. However, these dissolving MNs are often prepared using water‐soluble sugar or biodegradable polymer for rapid dissolution in intradermal fluid after insertion. Thus, it is difficult to encapsulate poorly water‐soluble vaccine components. In a recent study, Kim et al. developed amphiphilic triblock copolymer (Pluronic F127)‐based dissolving MNs encapsulated with OVA antigen and resiquimod (R848) (OVA/R848 MN) (**Figure** **4**A–C).^[^ ^100^ ^]^ Upon dissolution after intradermal administration, the Pluronic F127 could form in situ nanomicelles (NMCs) which encapsulated the released R848 and OVA simultaneously. On the one hand, the OVA and R848‐encapsulated NMCs could be captured and internalized by intradermal DCs, which then travelled to LNs. On the other hand, NMCs with a size between 30–40 nm are also optimal for effective trafficking to surrounding LNs, leading to an enhanced delivery efficiency of OVA and R848 to LNs‐resident DCs. Therefore, this dissolving MN‐based vaccine may synergistically enhance the DCs maturation and induce strong tumor‐specific immune response. In vivo studies showed that vaccination with this OVA/R848 MN could elicit both enhanced humoral and cellular antitumor immune response in mice. Importantly, treatment with this OVA/R848 MN vaccine not only significantly inhibit the progression of E.G7‐OVA tumor cells but also induced a strong memory antitumor immunity against tumor rechallenge, indicating the promising therapeutic effect of this OVA/R848 MN vaccine.

It should be noted that needle's size can be customized to enable transdermal delivery of vaccine components to subcutaneous region. For example, Ye et al. developed a transdermal MN patch loaded with tumor lysates combined with melanin and adjuvant (Figure 4D–H).^[^ ^101^ ^]^ After transdermal insertion into the skin, the whole tumor lysate and adjuvant underwent a gradual release from the MN patch and were taken up by skin‐resident DCs. In combination with the near‐infrared light (NIR) irradiation, melanin in the patch mediates the generation of heat, which further promotes tumor‐antigen uptake by DCs and leads to enhanced antitumor immunity. In addition, local heat causes the release of inflammatory cytokines that recruit immune cells, generation of immunogenic substrate such as extracellular heat shock proteins (HSPs), reactive oxygen species (ROS), antigen adjuvants, and some other danger signals that provoke the immune system. In vitro characterization of this fabricated MN patch demonstrated that the whole tumor lysate and melanin were successfully loaded into the MN patch with uniform loading and alignment. To evaluate the prophylactic effect, mice were challenged with B16F10 melanoma cells 10 days after vaccination. Mice receiving the MNs loaded with tumor lysate and granulocyte macrophage colony stimulating factor (GM‐CSF) as well as exogenous NIR irradiation showed long‐term survival with complete tumor regression in 87% of the treated mice. In sharp contrast, mice received only MNs loaded with tumor lysate and melanin but without NIR irradiation caused tumor regression in 13% of mice until day 30. Three days after combined vaccination, a 5.9‐fold increase in accumulated DCs [CD11c^+^, paired Ig‐like receptors of activating‐A/B^+^], indicating that loading of GM‐CSF in the MNs played an important role in the local recruitment of DCs. These stimulated DCs could then migrate to peripheral LNs to activate T cells‐based adaptive immune responses. Moreover, mice receiving the combined vaccination demonstrated much increased tumor‐infiltrated CD8^+^ T cells and mDCs populations compared to MN‐only group and control group. They also found that an eightfold increase in IgG titers in the serum of immunized mice as compared with mice in control group, indicating local immune activation was associated with systemic immune response. They further identified that local vaccination confers improved protection toward not only local tumors but also distant tumor from the NIR‐ and MN‐treated site. To determine the versatility of this proposed vaccination, they used a BRAFV600E‐mutated BP melanoma‐bearing mouse model and a triple‐negative breast cancer 4T1 carcinoma tumor‐bearing mouse model. Both tumor‐bearing mice model receiving vaccination with combined approach showed much prolonged survival time compared to that in MN‐only group and blank group, suggesting the capacity of NIR‐responsive MN vaccine to induce profound humoral and cellular antitumor immunity. All these studies validate the potential of MN patch‐based vaccine in targeting skin‐resident APCs and priming antitumor immunity. Moreover, a dose‐sparing effect of MN vaccine has been observed compared to the traditional intramuscular immunization, eliciting commensurate humoral, cellular and mucosal responses.^[^ ^102^ ^]^

#### 4.5.2 Injectable Hydrogel‐Based Modulation of Subcutaneous APCs

**Figure 5:**
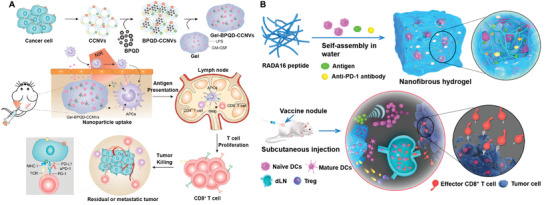
Hydrogel‐based vaccine for direct modulation of subcutaneous DCs. A) Schematic illustration of the preparation of Gel‐BPQD‐CCNVs vaccine and the mechanism for enhancing cancer immunotherapy. Reproduced with permission.^[^ ^105^ ^]^ Copyright 2019, American Chemical Society. B) Schematic illustration of the formation and mechanism of nanofibrous hydrogel‐based vaccine. Reproduced with permission.^[^ ^108^ ^]^ Copyright 2018, American Chemical Society.

Since a lot of DC populations are also residing in the subcutaneous network, delivering vaccine components to subcutaneous DCs represents the most common strategy. With the help of biomaterials, localized delivery of vaccine components to subcutaneous DCs population can be actively pursed. Among these biomaterials, biodegradable and soft hydrogel with injectable ability has been widely used for developing cancer vaccine due to the ability to allow persistent retention at vaccination site after s.c. injection and sustained release of antigen or adjuvant.^[^ ^103^ ^]^ For example, Bencherif et al. developed a sponge‐like macroporous cryogels containing GM‐CSF, CpG oligodeoxynucleotide (CpG ODN) as well as irradiated B16F10 cells.^[^ ^18^ ^]^ GM‐CSF is a cytokine that plays a critical role in immunomodulation, leukocyte development, proliferation and survival, and recruits the APCs including DCs.^[^ ^104^ ^]^ CpG ODN is a TLR agonist that can be used for DCs activation. The cryogels can be injected subcutaneously into mice to enable localized delivery of whole cell vaccine and immunomodulatory factors to DCs population in a spatiotemporally controlled manner. Vaccination with vaccine components‐loaded cyrogels induced a substantial increase of CD11c^+^, plasmacytoid as well as CD8^+^ DCs populations at vaccination site, spleen and draining LNs compared to blank cyrogels without any loading. More importantly, vaccination with these vaccine components‐loaded cryogels could elicit a robust CTL response in melanoma‐bearing mice, which significantly retarded tumor growth. Moreover, the cryogels vaccination could also ensure a long‐lasting and durable protective immune response, even at 4 months post‐vaccination. In another study, Ye et al. extracted the tumor cell membrane from surgically removed tumor to develop personalized cancer vaccine (**Figure** **5**A).^[^ ^105^ ^]^ It has been reported that using tumor cell membrane as vaccine source can reduce the contamination of intracellular materials and improve the specific response of the immune system.^[^ ^106^ ^]^ They coated the tumor cell membrane onto blank phosphorus quantum dot nanovesicle (BPQD‐CCNVs), which was then loaded into thermosensitive hydrogels containing GM‐CSF and lipopolysaccharide (LPS) to form hydrogel‐based vaccine (Gel‐BPQD‐CCNVs). BPQDs are photothermal materials with a high photothermal convention efficiency and good biodegradable property.^[^ ^107^ ^]^ Therefore, irradiation with NIR could lead to the generation of local heat, which can trigger the thermo‐responsive degradation of hydrogel. The encapsulated BPQD‐CCNVs could then be captured and internalized by subcutaneous DCs to stimulate their maturation. Meanwhile, the encapsulated GM‐CSF and LPS could also be released sustainedly to simultaneously recruit and activate DCs maturation, resulting in increased mDCs populations. These mDCs then travelled to surrounding LNs to present and activate tumor‐specific T cells. The antitumor studies showed that vaccination with Gel‐BPQD‐CCNVs could induce tumor‐specific CD8^+^ T cells response against either 4T1 or B16F10 tumor cells based on their cell membrane source, indicating the flexibility of this hydrogel in developing personalized vaccine. Moreover, combination of Gel‐BPQD‐CCNVs with *α*PD1 could significantly inhibit the surgical residual and lung metastasis.

In addition to targeting DCs for enhanced vaccine effect, hydrogel may also offer the opportunities to targeted modulation of activated T cells simultaneously. For example, Yang et al. developed a nanofibrous hydrogel containing a simple physical mixture of tumor antigen, *α*PD1 and exogenous DCs (Figure 5B).^[^ ^108^ ^]^ After s.c. injection, this nanofibrous hydrogel can synergistically enhance the antitumor immune response. On the one hand, the tumor antigens can be released to local host DCs to stimulate their maturation and the encapsulated exogeneous DCs could also be activated as well. On the other hand, the encapsulated *α*PD1 could bind the PD1 on activated T cells to pre‐block immune checkpoint on tumor cells. In vivo studies demonstrated that such hydrogel‐based vaccine could induce strong antitumor immune response in both prophylactic and therapeutic tumor models compared to adoptive transfer of DCs or antigen vaccine alone. Moreover, the combination with *α*PD1 further improves the antitumor immunotherapy effect. All these studies suggest the potential of injectable hydrogel as vaccine delivery platform for localized modulation of subcutaneous DCs population.

#### 4.5.3 Injectable Macroparticle‐Based Direct Modulation of Subcutaneous DCs Population

**Figure 6:**
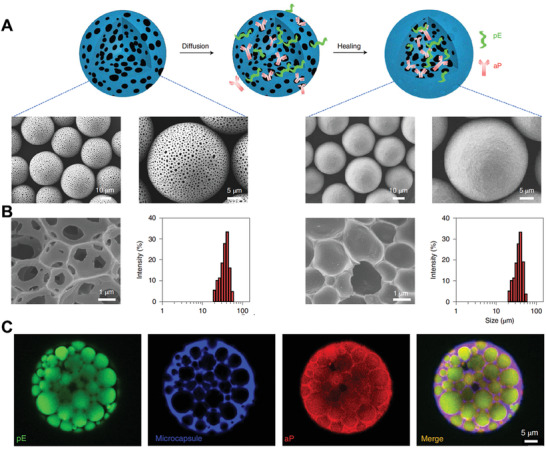
Macroparticle‐based vaccine. A) Schematic preparation of M(pE/aP) and corresponding SEM images. B) Size distribution of microspheres before and after the healing process. C) Representative fluorescent images of a microcapsule, in which the Eps8 peptide (pE) was labeled with fluorescein isothiocyanate, the microcapsule was labeled with Cy5, and the PD1 antibody (aP) was labeled with Cy3. Reproduced with permission.^[^ ^110^ ^]^ Copyright 2021, Nature Publish Group.

Similar effect can also be achieved by using microparticles which can retain at the vaccination site for a long time and enable release of antigen in a spatiotemporal manner.^[^ ^109^ ^]^ In a very recent study, Xie et al. developed biodegradable poly(lactic acid) (PLA) microparticle‐based therapeutic vaccine by co‐loading with a leukemia‐associated epitope peptide (pE) highly expressed in leukemia patients and *α*PD1.^[^ ^110^ ^]^ Owing to the porous structure and gentle infrared irradiation‐triggered self‐healing properties, the pE and aP could be encapsulated into microparticle with high loading efficiency (**Figure** **6**A–C). The resulting M(pE/aP) with a size distribution around 35 µm, which enable long‐term retention at vaccination site after s.c. injection. In vivo distribution study showed that free pE, free aP, or physical mixture of pE/aP which exhibited very rapid clearance (≈3 days) post‐vaccination. By contrast, M(pE/aP) could retain at the vaccination site as long as 5 weeks, thus enabling a long‐lasting release of pE and aP and much improved bioavailability. They found that vaccination with M(pE/aP) could lead to increased chemokines level and thus recruit APCs at vaccination site. Moreover, the released pE could be uptake by recruited DCs to stimulate their maturation, and the mDCs could then travel to LNs along with *α*PD1. As a result, vaccination with one single dose of M(pE/aP) could activate T cells in LNs and promote a much‐improved proliferation of CD8^+^ T cells. More importantly, this microparticle‐based platform can be flexibly optimized to load different epitope peptides to induce epitope‐specific T cell response. Thus, treatment with various epitope peptide‐based M(pE/aP) showed a promising immunotherapy effect in different leukemia‐bearing mice model derived from leukemia cell line, humanized cell line‐derived xenograft (PBMCs‐CDX), and humanized patient‐derived xenograft (PBMCs‐PDX). This study suggested that macroparticle can serve as promising platform for developing vaccine. By using FDA‐approved materials, microparticle‐based vaccine for clinical use can be actively pursed.

#### 4.5.4 Implantable Scaffold‐Based Direct Modulation of Subcutaneous DCs

Recently, implantable materials have emerged as another promising platform for in vivo modulation of host immune cells, including subcutaneous DCs. These implantable materials can be functionalized, pre‐loaded with tumor antigens, immunomodulatory agents or even cells before being physically inserted into a living host via minor invasive surgery. Usually, these implantable materials are characterized by macroscale size similar to a small tablet or pill and 3D porous structure, function as “artificial tertiary lymphoid structure”. After implantation into subcutaneous region or resected tissue space, they possess the ability to spatiotemporally control release of bioactive cargo, leading to the recruitment of immune cells into the large porous scaffold for further biological programming.^[^ ^111^ ^]^ A pioneer work conducted by Ali and Mooney in 2009 using macroscale poly(lactide‐*co*‐glycolide) (PLG) polymer scaffold loaded with tumor cell lysate, GM‐CSF and CpG‐ODN.^[^ ^112^ ^]^ Of which, the GM‐CSF acted as chemokine to recruit the subcutaneous DCs, tumor cell lysate was uptake by DCs, and CpG‐ODN acted as danger signal (adjuvant) to further DCs maturation. These mDCs then home to LNs to present and active tumor‐specific T cells. By providing these immunological cues, this PLG scaffold function as immunological niche that could in situ program specific DCs population for antitumor immunity. Although promising, this PLG scaffold required a surgical procedure, which may cause uncomfortable pain to patients. In a following study conducted by same group, they developed a high aspect‐of‐ratio, mesoporous silica rods (MSRs) that can be injected with needle into subcutaneous region and spontaneously assemble in situ to form 3D microporous scaffold to program host immune cell in vivo.^[^ ^113^ ^]^ After injection into the subcutaneous area, the MSRs‐formed scaffold can retain apparent at injected site for at least 2 weeks, which enabled a sustained release of GM‐CSF and CpG‐ODN. As described above, the localized and sustained release of GM‐CSF could recruit a much higher number of host DCs to the pores between the scaffold compared to pore‐filled silica microrods with same parameters or monolith type MSRs but lacking interparticle macropores. Subsequently, CpG‐ODN could promote the development of recruited DCs into mDCs, and these mDCs finally were capable of migrating out the MSRs into LNs for priming LN‐resident T cells. More importantly, when co‐loading with tumor antigen OVA, vaccination with MSRs scaffold led to a significantly high level of anti‐OVA IgG_2a_ and IgG_1_, which were the indicator of helper T cell 1 (T_H_1) and T_H_1 response, respectively. In comparison, vaccination with equivalent OVA, GM‐CSF and CpG‐ODN in bolus formulation elicited only a moderate and T_H_1‐skewed response and vaccination with bolus OVA alone led to very limited antibodies generation. Moreover, mice pre‐immunized with MSRs vaccine were found to significantly delay the progression of EG7.OVA lymphoma cells after subsequent challenge compared to control treatments, indicating the potent prophylactic tumor protection.

**Figure 7:**
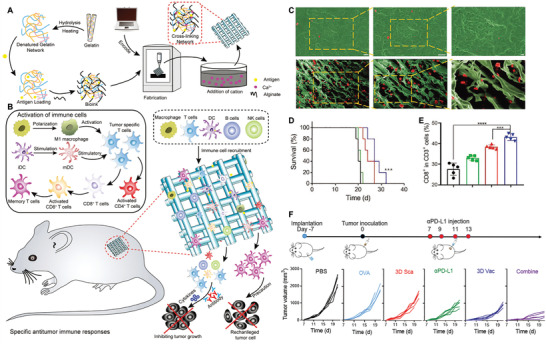
Implantable scaffold‐based vaccine. A) Schematic illustration of preparation of 3D printed scaffold vaccines. B) Schematic illustration of in vivo specific antitumor immune responses of implanted 3D‐scaffold vaccines. C) Representative SEM images of cell infiltration for hydrogel vaccine and 3D scaffold vaccine ex vivo. D) Survival curve of immunized mice after challenge with B16F10 tumor cells. E) The proportion of the tumor‐infiltrated CD8^+^ T cells in CD3^+^ T cells. F) Schematic illustration of scaffold vaccine combination with *α*PD‐L1 and individual tumor growth curves after different treatments. Reproduced with permission.^[^ ^115^ ^]^ Copyright 2021, Wiley‐VCH.

With advance of 3D printing technology, the use of 3D printing scaffold for biomedical application, such as bone engineering^[^ ^114^ ^]^ and cancer immunotherapy,^[^ ^115^ ^]^ has attracted increasing attention. Such technologies greatly improve the ability to produce a variety of complex and customized scaffold precisely, rapidly, economically, and with high reproducibility through layer‐by‐layer positioning of materials, bioactive molecules, and even living cells.^[^ ^116^ ^]^ Moreover, the 3D printing scaffold is often featured with well‐organized porous structure to enable the infiltration and influx of immune cells, which function as real lymphoid organ. By contrast, this accurate and repeatable porous structure is hard to be achieved by traditional hydrogel and chemical engineering approaches.^[^ ^117^ ^]^ Given to these advantages, personalized cancer vaccines based on 3D printing scaffold can be actively pursed. For example, Zhang et al. developed a porous and implantable 3D printing scaffold loaded with tumor antigen as prophylactic and therapeutic cancer vaccine (**Figure** **7**A,B).^[^ ^115^ ^]^ In vitro study showed that OVA‐loaded 3D scaffold vaccine could efficiently induce DC maturation. In vivo study showed that, after s.c. implantation, 3D scaffold vaccine could recruit a large number of DCs into the scaffold and promote their maturation (Figure 7C). In addition to DCs populations, this 3D scaffold can simultaneously attract other immune cells populations, including macrophages, NK cells, B cells, T cells, leading to the formation of microenvironment that enhance both humoral and cellular immune response. Compared to synthetic hydrogel vaccine with limited inner space, 3D scaffold vaccine could recruit much more immune cells into their porous space and thus efficiently suppress tumor growth. Furthermore, mice pre‐immunized with this 3D scaffold could significantly delay tumor progression when challenging with B16‐OVA cells compared to blank 3D scaffold, suggesting the promising prophylactic effect of this 3D scaffold (Figure 7D,E). Combination with 3D scaffold vaccine with *α*PD‐L1 further improved the anti‐tumor efficiency significantly (Figure 7F). Because of its good stability, easy manufacturing, biocompatibility, and excellent therapeutic and prophylactic effect, 3D scaffold vaccine may have promising future in clinical application.

#### 4.5.5 Nanovaccine‐Based Direct Modulation of Subcutaneous APCs

**Figure 8:**
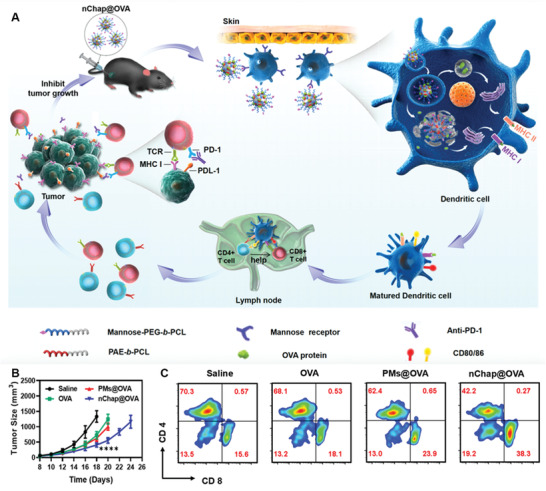
Nanovaccine‐based direct modulation of subcutaneous DCs. A) Schematic illustration of the mechanism of nChap@OVA for targeting subcutaneous DCs and inducing robust antitumor immunity. B) Average tumor growth curves of B16F10‐OVA tumor‐bearing mice treated with nChap@OVA and control formulation. C) Representative flow cytometry of analysis of the CD8^+^ and CD4^+^ T cells in the spleen from mice immunization with nChap@OVA and control formulation. Reproduced with permission.^[^ ^118^ ^]^ Copyright 2020, American Chemical Society.

Apart from the ability for targeting delivery to LNs and subsequent LNs‐resident APCs, nanovaccines modified with specific ligand also have the potential to target skin‐resident APCs, leading to their activation and subsequent trafficking to LNs. For example, Li et al. designed a mannose‐modified nanochaperone loaded with R848 and OVA (nChap@OVA) for enhancing antigen uptake by subcutaneous DCs (**Figure** **8**).^[^ ^118^ ^]^ After s.c. injection, such nChap@OVA with a size over 100 nm could specifically bind the MR on DCs surface, resulting in enhanced uptake. Upon entering the acidic lysosome of DCs, the hydrophobic microdomains of nChap@OVA underwent a transformation into hydrophilic chain with positive charge because of the pH‐responsive poly(*β*‐amino ester) PAE moiety, resulting in lysosome rupture and antigen escape into cytoplasm. Owing to both lysosomal and cytoplasmic delivery, antigen may be presented via either MHC‐I or MHC‐II pathway to activate both CD8^+^ and CD4^+^ T cells. In vitro study demonstrated that treatment with nChap@OVA led to a much higher CD11c^+^SIINFEKL^+^ percentage, suggesting the potential of nChap@OVA to stimulate DCs activation. Immunization with nChap@OVA significantly delay B16F10‐OVA tumor growth and prolong survival compared to OVA‐loaded PEGylated micelles (PMs@OVA), which was mainly owing to that nChap@OVA could induce higher antigen‐specific CD8^+^ and CD4^+^ T cells activation. Moreover, treatment with nChap@OVA and *α*PD1 exhibited a great inhibitory effect on tumor cell growth in established B16F10‐OVA mice model. These results together indicated that enhanced antigen uptake by subcutaneous DCs via mannose modification may also be a good option to improve antitumor vaccination efficiency. Meanwhile, this study also confirmed the notion described above that nanovaccines with a size over 100 nm tend to retain at vaccination site and be captured by subcutaneous DCs instead of trafficking to LNs.

## 5 Biomaterial‐Based T Cell Engineering

Since T cells play a direct role in governing antitumor immunity and killing tumor cells, adoptive T cell (ACT) therapies are a promising approach to treat cancers and shown unprecedented clinical success for the treatment of B‐cell acute lymphoblastic leukemia.^[^ ^10^ ^]^ Although promising, T cell‐based therapies are still facing several challenges which greatly restrict its broad implementation, such as lengthy manufacturing process, non‐persistent T cells, identification of optimal T cell subpopulation, and high cost.^[^ ^119^ ^]^ Therefore, targeted modulation of T cells using biomaterials both ex vivo and in vivo can be an alternative strategy, which has been actively pursed to amplify CTL response.

### 5.1 Artificial APCs for T Cells Expansion

Apart from the intrinsic limitation of CAR‐T therapy, such as on‐target and off‐target toxicities, undesirable immune‐related cytokine syndrome and the possibility of transformation, a lack of efficient procedures for expansion of cells also limited their broad implementation. Effective CAR‐T therapy relies on that these engineered T cells undergo efficient ex vivo activation, expansion, and differentiation through interaction with autologous natural APCs, such as DCs. However, the isolation and ex vivo stimulation of autologous DCs was proven time‐consuming and laborious. In a typical process, several months are required to produce therapeutic number (10^9^–10^11^) of tumor‐specific CTL. Meanwhile, the quality of ex vivo stimulated T cells can be variable due to the variable DCs generated ex vivo.^[^ ^120^ ^]^ Therefore, clinical translation of ACT therapy requires strategies that can manufacture cells efficiently and economically. To troubleshoot this issue, the use of biomimetic aAPCs has been developed as an alternative option for ex vivo activation of tumor‐specific CTL. aAPCs are 3D platforms that minimally integrate two signals for T cells activation: signal 1) peptide‐MHC complex to provide TCR specificity or activating antibodies for CD3 (*α*CD3, TCR stimulus) and signal 2) co‐stimulatory molecules (such as anti‐CD28 antibody, *α*CD28). In some cases, aAPCs also integrate a signal 3, cytokine (such as interleukin‐2, IL‐2), to further promote the long‐term proliferation of activated tumor‐specific CTL.^[^ ^121^ ^]^ Initial attempts that using paramagnetic microbeads (Dynabeads) functionalized with *α*CD3 and *α*CD28 to resemble aAPCs has been showed to stimulate robust human T cell expansion.^[^ ^122^ ^]^ Since then, *α*CD3/*α*CD28‐functionalized beads represent one of the most used synthetic aAPCs to expand T cells in multiple clinical trials.^[^ ^123^ ^]^ In addition to magnetic beads, a variety of biomaterials‐based aAPCs have been constructed recently, including polymeric‐based,^[^ ^124^ ^]^ lipid‐based,^[^ ^125^ ^]^ and inorganic‐based materials.^[^ ^113^ ^]^ These synthetic aAPCs can closely mimic the features of natural APCs for T cells activation while offer several advantages, such as long‐term storage, homologous presentation, sustained T cell activation, and low cost. Recently, 3D scaffold constructed by either carbon nanotubes^[^ ^126^ ^]^ or MSRs^[^ ^113^ ^]^ has emerged as efficient aAPCs for ex vivo T cell expansion given to the high aspect ratio and high surface area. For example, Fadel T.R. et al., developed an antigen‐coated bundled carbon nanotube, which was further combined with polymeric NPs containing magnetite and IL‐2. This carbon nanotube‐polymer scaffold can efficiently expand antigen‐specific CTL proliferation in vitro to a comparable number to clinical standards, while used thousand‐fold less soluble IL‐2. In a later study, Cheung A.S. et al., constructed an APC‐mimetic scaffold (APC‐ms) consisting of MSRs coated with supported lipid bilayers (MSRs‐SLBs) for mouse or human T cell expansion.^[^ ^127^ ^]^ One the one hand, the SLBs functionalized with T cell activation cue can activate T cells. One the other hand, the MSRs can enable sustained release of soluble IL‐2, which can facilitate the proliferation of activated T cells. Moreover, the MSRs‐SLBs demonstrated great feasibility to be functionalized with either *α*CD3/*α*CD28 for polyclonal T cell expansion or peptide‐MHC (pMHC)/*α*CD28 for antigen‐specific T cells expansion. In vitro studies showed that the APC‐ms stimulate two to tenfold higher polyclonal expansion of mouse primary and human T cells compared with commercial expansion Dynabeads. More specifically, APC‐ms induced over fivefold greater expansion of restimulated CD19 CAR‐T cells than Dynabeads, leading to even better therapeutic efficiency in a xenograft lymphoma model.

Apart from ex vivo expansion, nanoscale aAPCs also hold great potential for in vivo T cells expansion given to their enhanced lymphatic delivery post‐subcutaneous administration,^[^ ^128^ ^]^ which has been reviewed above. For example, Meyer et al. synthesized nanoellipsoidal PLGA‐based aAPCs can stimulate T cell activation in vivo following systemic administration.^[^ ^129^ ^]^ Compared to spherical aAPCs, nanoellipsoidal aAPCs showed enhanced ex vivo expansion of antigen specific CLT. In vivo study showed that nanoellipsoidal aAPCs not only elicit strong antitumor immunity but also possess better pharmacokinetic performance due to their resistance to mononuclear phagocytic system (MPS). Meanwhile, it has been reported that the particle size may play an important role in determining the efficiency of aAPCs in T cells activation. Hickey J.W. et al. systemically compared the effects of particle size on T cells activation based on superparamagnetic iron oxide NPs (SPIONs).^[^ ^130^ ^]^ Compared to aAPCs with a smaller size of 50 nm, aAPCs with a size of larger than 300 nm was more efficient to stimulate T cells activation, which was mainly owing to their ability to cluster around TCR and induce multivalent interactions in pMHC‐TCR interaction and co‐stimulatory interaction. Furthermore, they investigated the ligand density required for efficient T cells activation at different size. For larger size aAPCs, the required ligand density was much lower than that modified on smaller size aAPCs. Even with equivalent ligand density and particle concentration, the smaller size aAPCs are still less efficient than larger size aAPCs in inducing T cells activation, further supporting the notion that size is particularly important feature for designing nanoscale aAPCs rather than ligand density.

**Figure 9:**
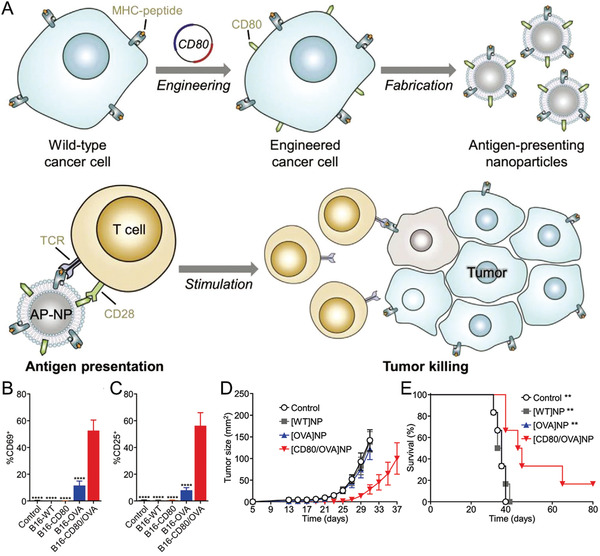
Tumor cell membrane‐coated NPs as biomimetic aAPCs. A) Schematic of engineered tumor cell membrane‐coated NPs as biomimetic aAPCs both ex vivo and in vivo. Expression of B) CD69 and C) CD25 by OT‐1 CD8^+^ T cells after incubation with [CD80/OVA]NPs and control NPs. D) Average tumor size and survival over time for the prophylactic efficiency evaluation in B16F10 melanoma mice model. Reproduced with permission.^[^ ^131^ ^]^ Copyright 2020, Wiley‐VCH.

Moreover, it should be noted that the conjugation of activation cue onto solid platform may lose some natural membrane functions, such as membrane fluidity and associated proteins, which also play a critical role in T cell activation. Biomimetic aAPC including cell‐based or cell membrane‐based aAPCs, has been explored as an alternative strategy. In one study, Jiang et al. developed a biomimetic aAPCs by coating tumor cell‐derived membrane onto NP for directly stimulating T cells (**Figure** **9**A).^[^ ^131^ ^]^ It has been reported that melanoma cell intrinsically expresses a certain degree of MHC‐I, which can enable the presentation of peptide epitopes from endogenous antigen to CD8^+^ T cells. To enable the presentation of pMHC complex to T cells in a more specific and immunostimulatory milieus, B16 wide type (B16‐WT) cells were genetically engineered to express both cytosolic OVA and co‐stimulatory marker CD80. The cell membrane derived from the engineered cells was then coated onto a polymeric NP core ([CD80/OVA]NPs) to stimulate tumor‐specific T cells expansion. Ex vivo study demonstrated that [CD80/OVA]NPs can efficiently elicit T cells activation as well as cytokine release in much higher level compared to B16‐WT cell membrane‐coated NPs ([WT]NPs) or only OVA‐expressed B16‐WT cell membrane coated NPs ([OVA]NPs) (Figure 9B,C). More importantly, [CD80/OVA]NP can also stimulate the activation of adoptive transferred T cells in vivo after i.v. injection, and significantly delayed the tumor growth (Figure 9D,E). The validation of [CD80/OVA]NPs for both in vitro and in vivo activation of tumor‐specific T cells provide a thinking to develop more efficient and feasible biomimetic aAPCs platform.

### 5.2 T Cell Engineering Ex Vivo

Another limitation of ACT therapy is the rapid decline in viability and function of transplanted cells. Therefore, after cell transfer, transplanted cells often rely on the concurrent administration of adjuvant drug (e.g., IL‐12 or IL‐15). However, these adjuvant drugs are required to be maintained at high and sustained level for efficiency, resulting in dose‐limiting cytotoxicity. To further enhance the therapeutic efficiency of transplanted cells in a more safety way, strategy involves the attachment of drug‐loaded nanocarriers to the membrane of transplanted cells ex vivo has been explored. For instance, Stephan et al. proposed a strategy that decorate cytokine‐loaded NPs onto the surface of T cell through thiol‐maleimide conjugation, thus enabling direct and sustained release of adjuvant drug to T cells.^[^ ^132^ ^]^ These cytokines such as IL‐21 and IL‐15 super agonist (IL‐15sa) are critical for T cell expansion. In vivo study revealed that the cytokine NP‐conjugated T cells concurrently administrated with T cells could elicit 4.9‐fold higher T cell proliferation than that T cells concurrently administrated with unencapsulated soluble cytokine. More importantly, cytokine NP‐conjugated T cells could significantly inhibit the tumor growth to even completed tumor clearance after systemic injection into melanoma‐bearing mice. In contrast, mice treated with only T cells or T cell with systemically administrated cytokine showed modest survival advantage. Two following studies conducted by same group used this strategy for delivering small molecular drug^[^ ^133^ ^]^ or protein^[^ ^134^ ^]^ to T cells. For instance, they developed lipid NPs loaded with NSC‐87877, a small molecular dual inhibitor of Shp1 and Shp2 which inhibit TCR activation in the synapse. Advanced prostate cancer‐bearing mice treated with NSC‐87877 NP‐conjugated tumor‐specific T cells demonstrated significant T cells activation at tumor site, as well as prolonged survival compared to those transplanted with only tumor‐specific T cells.^[^ ^133^ ^]^ To better control the drug release profile, they further reported that activated CD8^+^ T cells had a higher reduction rate than native T cells, which can be used as stimulus for drug release. Motivated by this finding, they first prepared a redox‐responsive protein nanogels (NGs) by cross‐linking the IL‐15sa with disulphide‐containing bis‐*N*‐hydroxy succinimide cross linker (NHS‐SS‐NHS). The disulphide cross‐linker was designed to be cleaved under the triggering of high level of reduction at T cell surface, leading to the controlled release of cytokines without any modification. To prevent the internalization of NGs by T cells, they further incorporate a small amount of anti‐CD45 antibodies into NGs. Meanwhile, NGs were also incorporated with a small quantity of poly(ethylene glycol)‐*b*‐poly‐l‐(lysine) (PEG‐PLL) to enable a slightly positive surface charge, which can enable the attachment of NGs onto T cell membrane through electronic interaction.^[^ ^134^ ^]^ In vivo study suggested that the reduction‐responsive NGs‐conjugated T cells could significantly expand T cells at tumor site to a 16‐fold higher level than T cell supported with systemic cytokines injection. Moreover, due to the high drug loading efficiency (>92% of dry weight), this NGs backpack allow eightfold more IL‐15sa to be administrated safely in mice compared to that with free cytokine. All these studies suggested the effectiveness of adjuvant drug‐loaded NP‐conjugated T cells in maintaining T cells activity and promoting T cell proliferation when transplanted into in vivo system.

More recently, mRNA has been explored as a promising strategy for inducing transient CAR expression because it can be translated without genomic integration.^[^ ^135^ ^]^ It should be noted that naked mRNA is easily to be degraded rapidly and is less likely to cross the cell membrane, effective delivery vectors or methods are thereby required to transfecting mRNA into T cells. Currently, electroporation has been widely used for promoting the mRNA into a variety of cells, including T cells.^[^ ^136^ ^]^ However, this method has several disadvantages, including membrane disruption, risk of losing cytoplasmic components, potential toxicity, and inconsistent membrane penetration.^[^ ^136^, ^137^ ^]^ Therefore, more reliable and safe transfecting platforms or methods are needed. Fortunately, functional biomaterials, such as ionizable lipid NPs (LNPs), have emerged as a good option for overcoming these challenges. For example, Billingsley et al. synthesized a library of 24 ionizable lipids and screened their mRNA delivery efficiency to T cell for engineering surface CAR.^[^ ^138^ ^]^ They selected a highest transfecting LNP formulation, C14‐4, for delivering CAR mRNA to human primary T cell. In vitro CAR expression study showed that CAR mRNA‐loaded LNPs induced 9.4‐fold higher expression in human primary T cells compared to untreated T cells, which was similar to that in electroporation‐treated T cells (tenfold higher). However, reduced cytotoxicity toward T cells was observed with LNP treatment compared to electroporation treatment. More importantly, in vitro coculture assay demonstrated that C14‐4 LNP‐engineered CAR‐T cells induced an equivalent killing activity toward Nalm‐6 acute lymphoblastic leukemia cells compared to electroporation‐treated T cells. The effectiveness of this study opens an avenue using LNP to transfect mRNA into T cells for CAR‐T cell engineering ex vivo, while the in vivo therapeutic potential needs more investigation.

### 5.3 In Situ Reprogramming of T Cells

**Figure 10:**
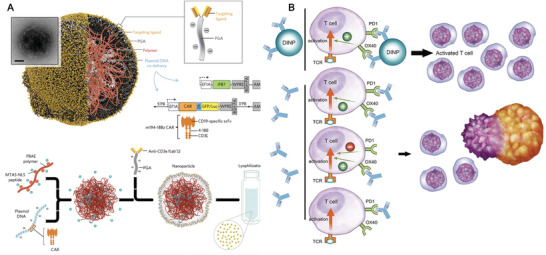
In situ T cell programming. A) Schematic diagram of structure of the in situ T‐cell‐engineered DNA NPs as well as their fabrication. Reproduced with permission.^[^ ^139^ ^]^ Copyright 2015, Nature Publish Group. B) Schematic illustration of DINP conjugated with *α*PD1 and *α*OX40 for binding to their targets simultaneously and enhancing combination immunotherapy. Reproduced with permission.^[^ ^140^ ^]^ Copyright 2018, Wiley‐VCH.

In addition to the capability of engineering T cells ex vivo, biomaterials have shown excellent potential for addressing these challenges by in situ reprogramming T cells. Unlike traditional ex vivo T cell engineering, Smith et al. proposed a method to rapidly reprogramming circulating T cells with tumor‐recognizing capabilities by encapsulating plasmid DNA encoding leukemia‐specific 194‐1BBz and transposase (iPB7) in PAE‐based NPs modified with T‐cell‐targeting anti‐CD3e f(ab’)2 fragments (**Figure** **10**A).^[^ ^139^ ^]^ The DNA‐carrying NPs can efficiently introduce leukemia‐targeting CAR genes into T cell nuclei, resulting in the expression of specific CAR and thus long‐term disease remission. Confocal imaging showed that the particles are rapidly internalized into cytoplasm, probably as a result of receptor‐mediated endocytosis. 30 h post‐transfection, the treated cells were detected with 194‐1BBz receptors on their surface (mean 3.8 ± 0.3% CAR^+^ T cells, NP:T cell ratio = 3 × 10^3^:1). Furthermore, in vivo anti‐tumor activities showed that 194‐1BBz‐carrying NP could induce substantial regression of leukemia tumor with a 58‐day improvement in survival. This effectiveness of in situ reprogramming of circulating T‐cell using T‐cell‐targeted NPs provide a practical, low‐cost, broadly applicable way to treat cancer.

In another study, Mi et al. developed dual immunotherapy NP (DINP) by decorating both *α*PD1 and antitumor necrosis factor receptor superfamily member 4 (*α*OX40) on polymeric NPs simultaneously (Figure 10B).^[^ ^140^ ^]^ *α*OX40 is an agonist antibody used for activating co‐stimulatory receptor to activate T cells. Their combination may exert a synergistic effect in boosting T cells activation and expansion, leading to improved antitumor effect. However, standard administration of these free antibodies may lead to only a subset of T cells binding to both *α*PD1 and *α*OX40 and thus activation of suboptimal T cells. By contrast, DINP can enable precise and simultaneous codelivery of *α*PD1 and *α*OX40 to T cells, leading to efficient T cells activation. DINPs could activate OT‐1 CD8^+^ T cells ex vivo to enhance the IFN‐*γ* release and tumor killing activity compared to treatment of mixed antibodies. In vivo study showed that DINP possessed a highest immunotherapeutic response against the xenograft B16F10 melanoma across all treatment groups with a cue rate of 30%. Meanwhile, durable memory antitumor immunity was induced in these cured mice when receiving tumor rechallenge. Moreover, in orthotopic 4T1 breast cancer model, DINP treatment also significantly controlled the tumor burden and prolonged the survival time of tumor‐bearing mice compared to other treatment groups. The excellent immunotherapeutic response was mainly owing to that DINP could specifically bind CD8^+^ T cells and stimulate their activation in situ after systemic injection. This study indicated that NPs‐based co‐delivery of immunotherapies may be a promising strategy for in situ T cells activation. Moreover, all these studies supported the great potential of in situ reprogramming T cells using biomaterials, while how to improve their delivery efficiency and specificity requires further investigation.

## 6 Targeting Delivery to TME for Reversing Immunosuppressive TME

TME is a complicated collection consisting of tumor cells, blood vessels, tumor‐infiltrating immune cells, tumor‐associated macrophages (TAMs), myeloid‐derived suppressor cells (MDSCs), cancer‐associated fibroblasts (CAFs), signaling molecules, and the ECM. These cells work together to alter the TME into an immunosuppressive milieu that inhibit the antitumor immune response while promotes tumor growth and metastasis.^[^ ^141^ ^]^ Therefore, targeting modulation of immunosuppressive TME to restore antitumor immunity is now regarded as the most popular strategy to boost immunotherapeutic effect while minimize off‐target side effect. To enable targeted modulation of immunosuppressive TME, delivering immunomodulatory molecules specifically to TME with enhanced accumulation is a prerequisite. Currently, a variety of delivery technologies have been widely used for targeting delivery of immunomodulatory agents to TME or TME‐resident cells, mainly relying on systemic delivery (systemically administrated nanomedicine) and localized delivery (implantable scaffold, injectable gel and MN).

### 6.1 Mechanism for Targeting Delivery to TME

Currently, delivering immunomodulatory agents are mainly relying on systemic administration and localized administration. For systemic delivery, ligand modification‐mediated active targeting and enhanced permeability and retention (EPR) effect‐based passive targeting are the major mechanisms that involved in targeting delivery of nanomedicines to TME or specific cells within TME. In contrast, localized delivery relies on the technology that enables direct delivery of drugs to TME without the need to travel through whole body system.

#### 6.1.1 Passive Targeting Delivery

Despite the fact that various non‐invasive administration of nanomedicines are now emerging, such as oral,^[^ ^142^ ^]^ pulmonary,^[^ ^143^ ^]^ nasal,^[^ ^144^ ^]^ most therapeutic nanomedicines for solid tumor treatment are administrated systemically. For decades, it has been considered that systemically injected NPs could preferentially home to tumor site through the EPR effect, which is resulted from the leaky tumor vasculature characterized by increased pore size and impaired lymphatic drainage, a process called passive targeting.^[^ ^145^ ^]^ On the one hand, blood vessels in TME have highly irregular architectures and are largely dysfunction when perfusing surrounding tissues compared to normal vessels.^[^ ^146^ ^]^ This is the result of excessive and rapid angiogenesis within TME, which is characterized by immature blood vessels that present a discontinuous epithelium and lack the basal membrane of normal vascular structures.^[^ ^145b,c^ ^]^ The pores between interendothelial cell junctions in the capillaries can reach sizes ranging from 10 to 1000 nm, leading to significantly higher vascular permeability and hydraulic conductivity.^[^ ^147^ ^]^ Thus, nanomedicines with size within the size range may have the opportunities to extravasate from blood vessel and penetrate into tumor interstitium. On the other hand, lymphatic vessels in TME are compressed by the proliferating cancer cells and stroma cells, particularly at the center of the tumor, leading to their collapse and impaired lymphatic flow.^[^ ^148^ ^]^ As a consequence, nanomedicines reaching the tumor interstitium cannot be cleared efficiently and thus be retained in the tumor interstitium.^[^ ^145^, ^149^ ^]^ However, the permeability of tumor between patients and between tumors in individual patient is highly heterogeneous, leading to heterogeneous EPR effect.^[^ ^150^ ^]^ Therefore, when designing EPR effect‐relying nanomedicine that aims to treat a specific tumor, the permeability profiles should be taken into consideration. In addition to the intrinsic tumor permeability, it has been reported that NP properties (for example, size, geometry, surface charge, surface chemistry, elasticity, stiffness) also play an important role in determining EPR effect. Therefore, understanding how these properties influence EPR effect may be of paramount importance to develop more effective nanomedicines for cancer immunotherapy. Given that these rationales for designing nanomedicine based on EPR effect have been well reviewed,^[^ ^145^, ^151^ ^]^ we will not review here.

#### 6.1.2 Active Targeting Delivery

Although passive targeting based on EPR effect is pronounced in preclinical models of solid tumors and is also observed in humans, the therapeutic benefits in patients with cancer remain heterogenous. This is largely owing to the pathophysiological heterogeneities (architecture and permeability of neovasculature) varying from tumor type, growth rate, microenvironment, and its localization.^[^ ^147^, ^152^ ^]^ Besides, passively‐targeted NPs deliver therapeutic agents nonspecifically into tumors, resulting in uptake by unintended target, reduced therapeutic effectiveness and increased off‐target side effect. Therefore, developing nanomedicines based solely on passive targeting strategy might not capitalize on their full potential benefit. By functionalizing targeting ligands onto NPs, they can specifically bind the receptors on the surface of tumor cells or tumor vasculatures upon entering into the TME and mediate selective delivery of therapeutic agents, a process called active targeting delivery.^[^ ^153^ ^]^ The interaction between receptors and ligand molecules allows enhanced anchoring of NPs onto the target cells or promotes NP internalization followed by the release of encapsulated drugs within intracellular compartment.^[^ ^154^ ^]^ The recognition ligands are a large class of endogenous or exogenous bioactive molecules including antibodies, proteins, peptides, nucleic acids, sugars, and small molecules. Target receptors can be proteins, sugars, lipid, or glycoprotein that overexpressed on diseased organs, tissues, cells, or subcellular domains. It has been reported that physiochemical properties of ligands, such as ligand size, ligand charge, ligand density, and ligand orientation might affect the efficacy of active targeting strategy both in vitro and in vivo.^[^ ^145c^ ^]^ Additionally, physiochemical properties governing passive transport and retention of NPs in tumors also affect actively‐targeted NPs.^[^ ^145c^ ^]^ Therefore, optimizing the physiochemical properties of both ligand and NPs is critical to design more efficient actively‐targeted NPs that may enable not only improved biodistribution and localization of the NPs within the tumor, but also improved internalization of NPs by cells.

#### 6.1.3 Localized Delivery

Although nanomedicines leveraging passive targeting and active targeting mechanisms have shown preclinical benefits over conventional drugs, the therapeutic benefits of most nanomedicines investigated in clinical trials remain unsatisfied.^[^ ^21a^ ^]^ Indeed, in a meta‐analysis study of 117 systemically administrated nanomedicines, some of these nanomedicines based on either passive targeting or active targeting mechanism only reach very low injected dose of 0.7%. This finding suggests the importance of rationally designing nanomedicine to further improve drug concentration at disease site as well as the need to develop alternative delivery strategy. It should be noted that different administration routes can lead to distinct delivery efficiency at tumor site. Unlike systemic administration, localized delivery technologies based on intratumoral injection, intratumoral implantation or transdermal patch may enable direct delivery of drugs to tumors without the need to travel through whole body system, leading to much higher accumulation of drugs in tumor sites. Fortunately, biomaterials, such as MN patch,^[^ ^155^ ^]^ injectable hydrogels,^[^ ^156^ ^]^ and implantable scaffold,^[^ ^157^ ^]^ have been widely explored to offer localized delivery of immunomodulatory agents. However, localized delivery technologies are generally more feasible for tumors that are easily accessible. In some case, metastatic tumor and haematological malignancies are hard to be accessed due to their unique pathogenesis and unpredictable location. Meanwhile, implantation often requires invasive surgery operation that may cause discomfort to patients. Therefore, when designing cancer immunotherapy based on localized delivery technology for specific cancer, the accessibility of tumor and patients’ willing should take into consideration.

### 6.2 NP‐Based Targeting Modulation of Tumor Cells

Since tumor cells can employ multiple mechanisms to suppress antitumor immunity and thus escape immune surveillance, the use of nanomedicine to target specific immunosuppressive mechanism has been regarded as a most popular strategy to restore antitumor immunity. Among these mechanisms, the immune checkpoint molecules, such as PD1 and PD‐L1, are highly relevant with immune resistance. Based on this notion, ICB using monoclonal antibodies (mAbs) has been widely investigated in both preclinical studies and clinical trials and demonstrated treatment benefits. However, the response rates of ICB antibodies vary among cancer patients and only a fraction of patients can benefit from this treatment. One of the major reasons is the poor delivery efficiency of antibodies to tumor site, which promotes the demands of more efficient delivery technologies for antibodies delivery.

#### 6.2.1 NP‐Based Delivery of Antibody for ICB Therapy

**Figure 11:**
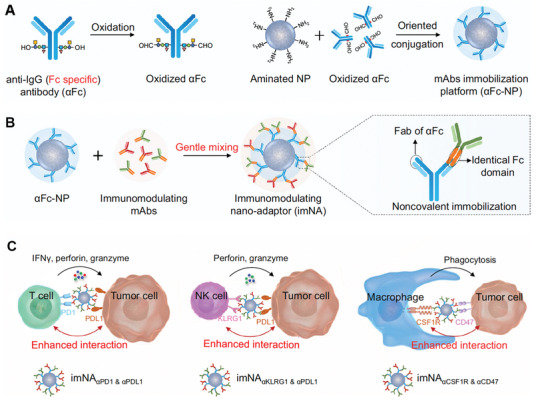
NPs conjugated with immunomodulatory mAbs for enhanced delivery efficiency and antitumor immunity. Schematic diagram of the preparation of A) *α*Fc‐NPs and B) imNA and their function to improve antitumor immunity after delivering to TME. C) The versatility of αFc‐NPs and superiority of imNAs were validated in T cell‐, natural killer cell‐ and macrophage‐mediated antitumor immune responses in multiple murine tumor models. Reproduced with permission.^[^ ^159^ ^]^ Copyright 2021, Nature Publishing Group.

Currently, the use of NPs for delivering antibodies by either surface conjugation or encapsulation has showed improved delivery efficiency. For example, Bu et al. conjugated *α*PD‐L1 onto hyperbranched, multivalent poly(amidomine) dendrimers to prepare G7‐*α*PD‐L1. G7‐*α*PD‐L1 was designed to bind specifically to PD‐L1 on tumor cells after systemic administration, leading to the blockade of PD‐L1 and improved T cells activity.^[^ ^158^ ^]^ In vitro binding kinetics assays exhibited that G7‐*α*PD‐L1 possessed enhanced binding avidity to PD‐L1 protein compared to free *α*PD‐L1. To test the in vivo binding kinetic of G7‐*α*PD‐L1, they further optimized the ratio of antibodies per dendrimer to 9:1 to ensure their selective and sufficient tumor accumulation. In vivo imaging demonstrated that a 2.5‐fold higher intensity of G7‐*α*PD‐L1 than free *α*PD‐L1 in tumor site post‐72 h administration. This finding suggested that nanocarrier can not only improve the binding avidity of *α*PD‐L1 toward PD‐L1 but also enhance their tumor accumulation. However, the in vivo antitumor immunotherapy efficacy of G7‐*α*PD‐L1 needs more investigation. In a recent study, Jiang et al. proposed a combination strategy by immobilizing two types of mAbs onto a single NP to integrate their individual function (**Figure** **11**).^[^ ^159^ ^]^ Of which, one mAb was designed to bind against effector T cells and the other mAb was designed to bind against tumor cells. Therefore, such engineered formulation (immunomodulating nano‐adaptor, imNA) may associate with both immune cells and tumor cells to bridge them together like “adaptor” while maintaining the immunomodulatory properties of original mAbs after delivering to TME. To develop imNA, they first build up a versatile antibody immobilizing platform by conjugating anti‐IgG (Fc specific) antibody (*α*Fc) onto the surface of NPs (*α*Fc‐NPs). Subsequently, *α*Fc‐NPs could recognize and immobilize any mAbs containing the Fc fragment. In vitro studies confirmed that *α*Fc‐NPs could efficiently immobilize one or two mAbs (*α*PD‐L1 and *α*PD1) simultaneously after a gentle mixing and short incubation. Considering the different populations of immune cells and tumor cells as well as their targets, predetermined ratios of *α*PD1/*α*PD‐L1 can be achieved by optimizing the feeding amounts of *α*PD1 and *α*PD‐L1, suggesting the well‐controlled immobilizing manner. Besides, the immobilization of mAbs onto NPs showed similar binding affinity to their targeted protein compared to free compartment. In vitro and in vivo studies confirmed that *α*Fc‐NPs immobilized with *α*PD‐L1 and *α*PD1 (imNA_*α*PD1&*α*PD‐L1_) could enhance T cell‐mediated cytotoxicity by simultaneously binding PD1 on T cells and PD‐L1 on tumor cells compared to physical mixing of *α*PD‐L1 and *α*PD1. More importantly, imNA_*α*PD1&*α*PD‐L1_ demonstrated a much enhanced accumulation at tumor site as well as improved antitumor CD8^+^ T cells effect in both B16F10 melanoma and 4T1 breast tumor model. In addition to *α*PD‐L1 and *α*PD1, *α*Fc‐NPs could also be used for immobilizing other mAbs that targeting immunomodulatory molecules expressed by tumor cells and other immune cells (e.g., NK cells and macrophages), such as anti‐KLRG1 (killer‐cell lectin‐like receptor G1) antibody *α*KLRG1 with *α*PD‐L1 or anti‐CSF1R (colony stimulating factor 1 receptor) antibody (*α*CSF1R) with anti‐CD47 antibody (*α*CD47). These studies validated that conjugating immunomodulatory mAbs onto NPs to enhance delivery efficiency and improve antitumor immunity present a promising strategy.

#### 6.2.2 NP‐Based Delivery of siRNA and Small Molecular Inhibitor for ICB Therapy

**Figure 12:**
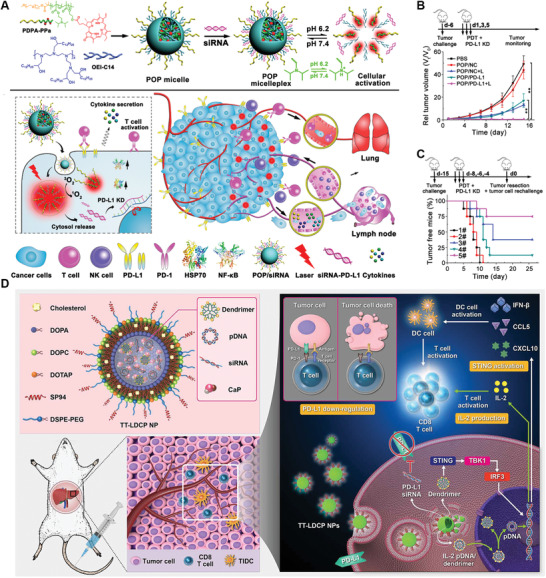
NPs for delivering PD‐L1 siRNA. A) Schematic illustration of preparation of POP micelleplex for loading PD‐L1 siRNA and its function to enhance photodynamic immunotherapy effect. B) Tumor growth inhibition in B16F10 melanoma‐bearing mice after different treatment. C) Tumor recurrence prevention after different treatments. Reproduced with permission.^[^ ^160^ ^]^ Copyright 2016, American Chemical Society. D) Schematic diagram of the mechanism of immunogene therapy by TT‐LDCP NPs containing siRNA against the immune checkpoint PD‐L1 and pDNA encoding the immunostimulating cytokine IL‐2. Reproduced with permission.^[^ ^163^ ^]^ Copyright 2020, American Association for the Advancement of Science.

Recently, the rapid advance of RNA interference (RNAi) therapy has offered the opportunity to develop alternative ICB therapy strategy by genetically intervention of PD‐L1 expression. However, the rapid degradation and poor stability of siRNA pose challenges for their delivery and wide implementation. Fortunately, the emerging of ionizable LNPs and polycation micelles with positively charged moiety enable efficient encapsulation of siRNA and protect them from degradation, leading to prolonged circulation time. Moreover, these NPs can enable high delivery efficiency of siRNA to specific cells and achieve rapid endosomal/lysosomal escape to cytoplasm to exert its function. For instance, Wang et al. designed a pH‐sensitive micelleplex (POP) consisting of a pH‐sensitive diblock copolymer PEG‐*b*‐poly(diisopropanolamino ethyl methacrylate‐*co*‐hydroxyethyl methacrylate) (PEG‐PDPA) conjugated with pheophorbide A (PPa, a photosensitizer) and a polycation 1,2‐epoxytetradecane alkylated oligoethylenimine (OEI‐C14) (**Figure** **12**A).^[^ ^160^ ^]^ The PDPA is a pH‐sensitive diblock copolymer of which the tertiary amines can be easily protonated in weakly acidified microenvironment.^[^ ^161^ ^]^ The OEI‐C14 is an amphiphilic polycation with strong siRNA binding affinity and proton sponge capability to trigger endosomal/lysosomal escape.^[^ ^162^ ^]^ In vivo studies confirmed that POP had a strong siRNA binding affinity and could enable rapid cytosolic release of siRNA. They further encapsulated PD‐L1 siRNA into this micelleplex (POP‐PD‐L1) and showed that POP‐PD‐L1 could significantly suppress PD‐L1 expression in B16F10 cells in a dose‐dependent manner. In vivo distribution study showed that POP‐PD‐L1 could efficiently deliver to tumor site via EPR effect. Moreover, they found that treatment with POP‐PD‐L1 significantly suppressed the PD‐L1 expression in tumor in vivo and combination with laser further enhanced the PD‐L1 silencing effect. Importantly, treatment with POP‐PD‐L1 greatly delay the tumor growth compared to POP loaded with scramble siRNA, suggesting the therapeutic potential of POP‐PD‐L1. Further combination with laser irradiation demonstrated an almost complete tumoricidal effect in B16F10 melanoma‐bearing mice and significantly prolonged survival, suggesting the great synergistic antitumor effect of PD‐L1 knockdown and photodynamic therapy (PDT) (Figure 12B,C).

In a recent study, Huang et al. developed tumor‐targeting lipid‐dendrimer‐calcium‐phosphate NPs (TT‐LDCP NPs) encapsulated with thymine‐functionalized polyamidoamine (PAMAM) dendrimer, PD‐L1 siRNA, and plasmid DNA encoding immunostimulating IL‐2 cytokine (IL‐2 pDNA) to reverse immunosuppressive TME and activate antitumor immunity simultaneously (Figure 12D).^[^ ^163^ ^]^ Such immunogenic TT‐LDCP NPs possess multiple functionalities, including i) a tumor‐targeting peptide (SP94) that ensure targeting delivery to hepatocellular carcinoma (HCC) tumor site and enhance following uptake by HCC cells as well as intracellular delivery of the siRNA/pDNA; ii) a pH‐sensitive calcium‐phosphate (CaP) core that enable rapid endosome escape and enhanced release of siRNA to silence PD‐L1 expression; iii) thymine‐functionalized PAMAM dendrimer further promote endosomal/lysosomal escape and nuclear entry of pDNA to encode IL‐2 gene; iv) thymine‐functionalized PAMAM dendrimer could also act as immunological adjuvant to activate stimulator of interferon genes (STING)‐cyclic GMP‐AMP synthase (cGAS) pathway to improve cellular immunity. Both in vitro and in vivo studies demonstrated that TT‐LDCP NPs efficiently deliver PD‐L1 siRNA and IL‐2 pDNA into HCC cells with a high transfection efficiency compared to non‐targeted LDCP NPs and TT‐LCP without dendrimer. Therefore, treatment with PD‐L1 siRNA and IL‐2 pDNA‐loaded TT‐LDCP NPs efficiently silenced the PD‐L1 expression while increased IL‐2 expression in HCC cells, synergistically leading to enhanced infiltration of CD8^+^ T cells into TME. They further evaluate the antitumor immunotherapy effect of PD‐L1 siRNA and IL‐2 pDNA‐loaded TT‐LDCP NPs in orthotopic HCC tumor model. Treatment with PD‐L1 siRNA and IL‐2 pDNA‐loaded TT‐LDCP NPs significantly suppressed the tumor growth and metastasis compared to that treated with TT‐LDCP loaded with control siRNA and HCC vaccine. Moreover, the combination of PD‐L1 siRNA and IL‐2 pDNA‐loaded TT‐LDCP NPs with HCC vaccine further improve the tumoricidal effect, suggesting that these immunogenic NPs could efficiently reverse immunosuppressive TME to favor antitumor immunity. These studies validate that RNAi can serve as a promising candidate for ICB therapy by using well‐designed NPs delivery platform. In addition to downregulation of PD‐L1 expression using RNAi technology, metformin (MET), a widely used small molecule drug for the treatment of type 2 diabetes, has also been reported to involve in endoplasmic‐reticulum degradation of PD‐L1.^[^ ^164^ ^]^ Therefore, targeting delivery of MET to tumor cells and enhance its uptake may be a good alternative strategy to sensitize other therapy. For instance, our group has developed an enzyme‐cleavable self‐delivery NPs (MA‐pepA‐Ce6 NPs) by conjugating acid‐sensitive MET prodrug with photosensitizer (chlorin e6, Ce6) through matrix metalloproteinase‐2 (MMP‐2) cleavable peptide (GPLGVRGDK, pepA).^[^ ^165^ ^]^ After delivering to tumor site via EPR, MA‐pepA‐Ce6 NPs could be cleaved by overexpressed MMP‐2 to release VRGDK‐Ce6. VRGDK‐Ce6 could further bind specifically to integrin *α*_v_ *β*_3_ on tumor cells, leading to enhanced internalization. Meanwhile, MET prodrug could be released under the triggering of acidic pH condition of TME to suppress PD‐L1 expression and augment antitumor immune response induced by PDT. In vitro and in vivo studies showed that MA‐pepA‐Ce6 NPs treatment led to an obvious downregulation of PD‐L1 in 4T1 cells compared to other treatments. Moreover, treatment with MA‐pepA‐Ce6 NPs showed a significant suppression of tumor growth in 4T1 tumor mice model compared to PBS treatment, suggesting that downregulation of PD‐L1 may delay tumor progression. Further combination with laser irradiation enhanced the antitumor efficiency and alleviates pulmonary metastasis, indicating that downregulation of PD‐L1 sensitized PDT‐induced immunogenic cell death (ICD) effect.

#### 6.2.3 NP‐Based Delivery of Antagonist against Immunosuppressive Molecules

**Figure 13:**
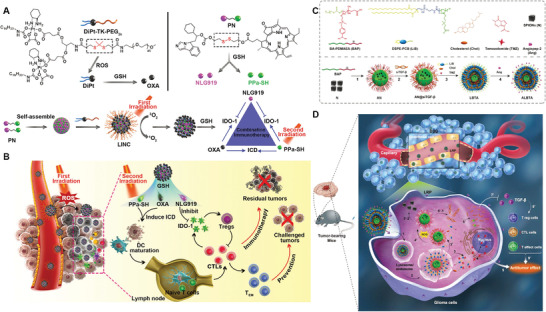
NPs for targeting inhibition of immunosuppressive small molecule. A) Schematic illustration of the fabrication of the LINC and B) mechanism for improved drug delivery and chemoimmunotherapy by eliciting tumor immunity and overcoming immunosuppressive TME. Reproduced with permission.^[^ ^168^ ^]^ Copyright 2019, Wiley‐VCH. C) Schematic illustration of the preparation of the ALBTA and D) mechanism for glioblastoma‐targeting delivery, drug release, improving antitumor immunity, and reversing immunosuppressive TME. Reproduced with permission.^[^ ^173^ ^]^ Copyright 2018, Wiley‐VCH.

Small molecules with immunosuppressive function have also been explored as a potential target, such as IDO. IDO can catalyze the degradation of tryptophan (Trp) and accumulation of kynurenine (Kyn), which could impair the survival and activity of CTLs cells.^[^ ^166^ ^]^ Meanwhile, Kyn suppresses the antitumor immunity of CTLs by activating regulatory T cells (Treg) and MDSCs.^[^ ^167^ ^]^ Therefore, targeting inhibition of IDO may be a promising strategy to reverse immunosuppressive TME and restore effector CTLs activity. For example, Feng et al. developed a TME‐activable binary corporative prodrug NP (BCPN) to improve immunotherapy by synergistically modulating the TME. BCPN was composed of an acidic and reduction dual‐responsive oxaliplatin (OXA) prodrug for triggering ICD effect and a reduction‐activatable homodimer of NLG919 for inhibiting IDO. Upon entering TME, the outside PEG shell could be cleaved in response to acidic pH condition, leading to charge reversion from negative to positive, which was beneficial for penetrating into deep tumor site. In vitro studies proven that treatment with acid‐sensitive BCPNs (^AS^PN) induced apparent ICD effect in tumor cells and DC maturation. Meanwhile, ^AS^PN pre‐treated in pH 6.5 condition showed enhanced inhibition efficiency against IDO compared to free NLG919 and ^AS^PN pre‐treated in pH 7.4 condition. In vivo antitumor studies showed that treatment with ^AS^PN significantly delayed the tumor growth in 4T1 tumor mice model and effectively prevented the pulmonary metastasis compared to other treatments, which was mainly owing to the synergistic antitumor effect of OXA‐induced ICD and NLG919‐mediated IDO inhibition. In a following study conducted by same group, they further combined the treatment modality (OXA prodrug and NLG919) with PDT by constructing a light‐inducible nanocargo (LINC) for cancer treatment. LINC is composed of reduction‐responsive photosensitize PPa, NLG919 and light‐activable prodrug of OXA (**Figure** **13**A,B).^[^ ^168^ ^]^ Upon receiving first‐wave NIR irradiation, the PPa component of LINC could generate ROS to trigger the cleavage of PEG shell, thus promoting tumor retention and deep tumor penetration. When receiving second‐wave NIR irradiation, LINC efficiently elicited an efficient ICD effect in tumor cells, leading to the infiltration of CTLs. Meanwhile, NLG919 could reverse the immunosuppressive TME to further enhance ICD effect by inhibiting IDO activity. In vivo studies further validated that NLG919 significantly inhibited the IDO activity in 4T1 breast tumor model and thus suppressed the Treg differentiation. Moreover, synergistic delivery with NLG919 significantly improved the antitumor effect of LINC compared to that without NLG919, supporting the importance of inhibiting IDO activity could boost immunotherapy effect.

TGF‐*β*, a soluble small molecule, also play an important role in tumor initiation and progression, functioning as both a suppressor and a promoter.^[^ ^169^ ^]^ It has been reported that genetic depletion or downregulation of TGF‐*β* signaling pathway often results in a more malignant phenotype in various types of tumors.^[^ ^170^ ^]^ This is due to that TGF‐*β* suppresses tumor initiation and early development via inhibiting cell cycle progression, inducing cell apoptosis and inhibiting expression of growth factors and cytokines.^[^ ^171^ ^]^ However, in most case, TGF‐*β* is known to be pro‐oncogenic and is often produced in large quantities by many tumor types. Several mechanisms are involved in the tumor promotion by TGF‐*β*, including dysregulation of cyclin‐dependent kinase inhibitor, alternation of cytoskeletal architecture, promotion of extracellular matrix formation, as well as compromising immune surveillance.^[^ ^171^ ^]^ It has been reported that high level of TGF‐*β* in TME inhibits T cells and B cells proliferation and promote Treg differentiation, thus promoting immunosuppressive TME.^[^ ^172^ ^]^ Therefore, downregulation of TGF‐*β* may be a promising way to reverse immunosuppressive TME to favor cancer immunotherapy or chemotherapy. For example, Qiao et al. designed a nanotheranostic system with dual targeting and ROS response for intracranial glioblastoma treatment (Figure 13C,D).^[^ ^173^ ^]^ This nanotheranostic system is consisting of several components including poly[(2‐acryloyl)ethyl(p‐boronic acid benzyl)diethylammonium bromide] (BA‐PDEAEA, BAP), zwitterionic lipid distearoyl phosphoethanol‐aminepolycarboxybetaine (DSPE‐PCB), cholesterol, angiopep‐2, temozolomide (TMZ), TGF‐*β* siRNA, and SPIONs, termed as (Ang‐LiB(T+AN@siTGF‐*β*), ALBTA). BAP is a positively charged and ROS‐responsive polymer used for binding and controlled release of TGF‐*β* siRNA, DSPE‐PCB is a polycation that enables endosome escape of siRNA and angiopep‐2 is dual‐targeting ligand used for crossing blood‐brain barrier and targeting glioblastoma cells. In vitro studies displayed that ALBTA significantly enhanced cellular uptake by GL261 glioblastoma cells and effectively promoted endosome escape of TGF‐*β* siRNA, leading to a downregulation of TGF‐*β* expression. In vivo studies showed that treatment with ALBTA induced a much higher proliferation of CD8^+^ and CD4^+^ T cells in spleen while greatly reduced the Treg differentiation compared to free TGF‐*β* siRNA and other treatments, suggesting that downregulation of TGF‐*β* expression favor the prime of antitumor immunity.

### 6.3 NP‐Based Targeting Modulation of TAMs

TAMs are a complex and heterogeneous population of immune cells in the tumor stroma and are a major source of secreted growth factors, cytokines, and chemoattractants.^[^ ^174^ ^]^ TAMs are actively recruited to tumors and play an important role in tumor progression, metastasis, and therapeutic resistance.^[^ ^175^ ^]^ Despite the existence of both pro‐inflammatory M1‐type and anti‐inflammatory M2‐type macrophages, TAMs predominately exhibit M2‐type function that contributes mainly to the development of an immunosuppressive, pro‐tumoral microenvironment.^[^ ^175^, ^176^ ^]^ TAMs can also facilitate the invasion and angiogenesis of aggressive tumor cells. Therefore, targeting modulation of TAMs is a promising therapeutic approach against cancer. Current TAMs‐targeted modulation strategies under development mainly focus on: 1) inhibition of monocyte/macrophage recruitment; 2) depletion of M2‐like macrophages; or 3) re‐polarization of pro‐tumoral M1‐like phenotype to tumoricidal M1‐like phenotype.^[^ ^177^ ^]^ It has been reported that CSF1 secreted by tumor cells bind CSF1R on macrophage membrane, resulting in the activation of downstream signaling pathway responsible for the polarization of TAM to immunosuppressive phenotype.^[^ ^178^ ^]^ Meanwhile, CSF1‐CSF1R axis also plays a critical role in promoting the myeloid progenitors into macrophages, monocytes, and DCs.^[^ ^179^ ^]^ Therefore, blocking CSF1‐CSF1R axis may reduce TAM proliferation and abundance in TME and facilitate infiltration of tumor‐specific T cells, thus enhancing the efficacy of ICB therapy of cancer.^[^ ^180^ ^]^ Currently, mAbs and small molecular inhibitors targeting the CSF1‐CSF1R have been used for cancer immunotherapy and some of them are being investigated in clinical trials. Among these, BLZ‐945, a hydrophobic small molecular drug, is a highly selective CSF1R inhibitor. Shen et al. developed a pH‐responsive immunostimulatory nanocarrier loaded with BLZ‐945 and platinum (^BLZ‐945^SCNs/Pt) that could simultaneously target TAM and tumor cells for combination therapy.^[^ ^181^ ^]^ After passive delivery to perivascular region of TME, ^BLZ‐945^SCNs/Pt could undergo a rapid structure collapse into small NPs under the triggering of acidic pH, leading to the simultaneous release of Pt‐prodrug and BLZ‐945. BLZ‐945 could be then taken up by the TAM to cause TAM depletion and Pt‐conjugated small NPs penetrated into deep tumor tissues and release active Pt intracellularly to kill tumor cell, leading to a synergistic tumoricidal effect. In vivo study further validated that treatment with ^BLZ‐945^SCNs/Pt greatly decrease the TAM population and increase CD8^+^ T cells, leading to significant suppression of tumor growth in 4T1 breast tumor‐bearing mice model.

Currently, polarization of TAM represents the most popular strategy for enhancing immunotherapeutic effect. For example, Rodell et al. found that R848, an agonist of TLR7/8 identified in a morphometric‐based screen, is a promising promoter to re‐polarize the M2‐like TAMs to M1‐like TAMs and showed similar effectiveness to LPS/IFN*γ* treatment.^[^ ^182^ ^]^ To deliver R848 specifically to macrophage, they further developed *β*‐cyclodextrin (CD) NPs loaded with R848 (CDNPs‐R848) by guest–host complexation. In vivo biodistribution demonstrated a highest accumulation of CDNPs in tumors (94.9 ± 1.9% ID per g tissue) following by draining LNs (93.0 ± 6.6% ID per g tissue) after i.v. injection, suggesting the tumor‐targeting ability of CDNPs. By utilizing a recently described receptor mouse wherein TAMs are readily detectable through MerTK^GFP/+^ expression, they observed the rapid vascular distribution of VT680‐labeled CDNPs (CDNP‐VT680) adjacent to and throughout the tumor and accumulation within GFP^+^ perivascular macrophages within 60 min. At 24 h post‐injection, CDNPs were cleared from the vasculature and had accumulated within TAMs throughout the tumors, indicating the precise TAMs‐targeting efficiency. More importantly, treatment with CDNPs‐R848 significantly inhibit tumor growth rates and prolonged the survival of MC38‐bearing mice compared to other treatments, indicating the potential of M1‐like TAMs in suppressing tumor growth. Moreover, combination with *α*PD1 exerted a synergistic effect in inhibiting tumor growth, leading to remarkable tumor shrinkage as well as stabilized and homogenous immune response. Other TLR agonists, such as IMQ, IL‐10, *α*CD40, IFN‐*γ*, have been encapsulated into or integrated onto NPs for TAM polarization. Apart from TLR agonist, hydroxychloroquine (HCQ), a known autophagy inhibitor, has recently been reported with the ability to induce the polarization of M2‐like TAM to M1‐like TAM.^[^ ^183^ ^]^ Based on this, our group has recently developed a furin‐responsive aggregable NPs loaded with doxorubicin (DOX) and HCQ (AuNPs‐D&H‐R&C) for combination treatment against breast cancer.^[^ ^184^ ^]^ After systemic administration, AuNPs‐D&H‐R&C could passively deliver to tumor site via EPR effect and formed aggregates under the triggering of overexpressed furin, leading to an increased size. These aggregates with increased size in turn blocked the back‐flow from tumor interstitium to bloodstream, further enhancing the retention of DOX and HCQ at tumor site. In vitro and in vivo studies showed that treatment with AuNPs‐D&H‐R&C increased the percentage of M1‐like TAM while decreased the percentage of M2‐like TAM. Furthermore, treatment with the furin‐responsive NPs significantly inhibited the tumor growth through a combination effect of chemotherapy, autophagy inhibition, and TAM polarization. Moreover, the endogenous ROS,^[^ ^185^ ^]^ Fe^3+^/Fe^2+[^ ^186^ ^]^ have been reported with the ability to induce TAM polarization from M2 phenotype toward M1 phenotype.

**Figure 14:**
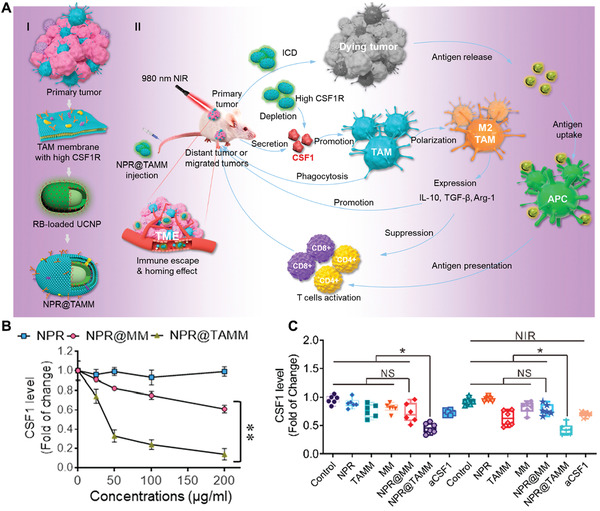
NPs‐based targeted modulation of TAMs. A) Schematic illustration of construction of NRP@TAMM and its function for improved photodynamic immunotherapy. B) In vitro ELISA analysis of CSF1 content in serum after incubation with NRP@TAMM and control NPs. C) ELISA analysis of CSF1 content in serum from tumor‐bearing mice treated with NRP@TAMM and control formulations. Reproduced with permission.^[^ ^187^ ^]^ Copyright 2021, American Chemical Society.

In a recent study, Chen et al. reported a TAM‐derived membrane (TAMM) coated upconversion NPs loaded with photosensitizer (NRP@TAMM) for photodynamic immunotherapy (**Figure** **14**A–C).^[^ ^187^ ^]^ This TAMM is introduced to not only exert superior antigen‐homing affinity capacity after systemic injection but also to mimic the cell source to scavenge the CSF1 secreted by tumor cells in TME, leading to the polarization of TAM. Meanwhile, the combination with PDT may further enhance the tumoricidal effect. In vitro and in vivo studies exhibited that NRP@TAMM could effectively decrease CSF1 level compared to other treatments. Meanwhile, combination treatment with NRP@TAMM plus NIR irradiation led to decreased M2‐like TAMs population while increased M1‐like TAMs population. More importantly, treatment with NRP@TAMM plus NIR irradiation significantly inhibited the tumor growth in both primary tumor and distant tumor model, as well as suppressed lung metastasis compared to other treatment, suggesting the great potential of synergistic therapy of TAM polarization with PDT. All these studies validate the potential of TAMs‐targeting strategy in boosting cancer immunotherapeutic effect.

**Figure 15:**
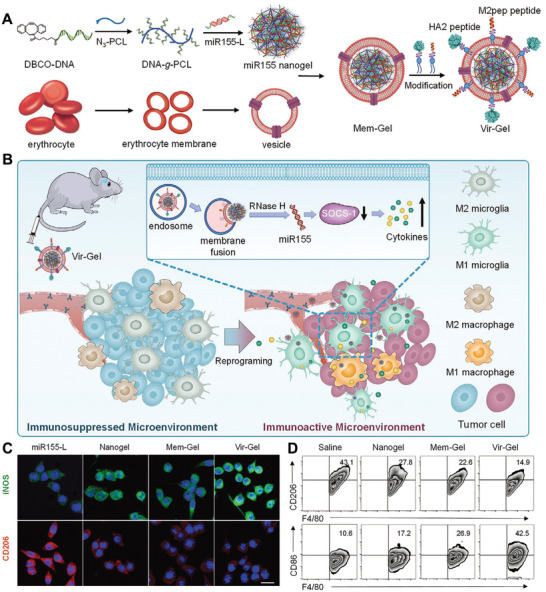
NPs‐based targeted modulation of microglia. A) Schematic illustration of preparation of peptide‐modified and erythrocyte membrane‐coated Vir‐Gel. B) Schematic illustration of mechanism of Vir‐Gel in polarizing anti‐inflammatory M2‐phenotype microglia to a pro‐inflammatory M1‐phenotype within TME of glioma. C) Immunofluorescence staining of M2‐like BV‐2 cells with iNOS (M1 marker) and CD206 (M2 marker) after pre‐treatment with Vir‐Gel and control groups for 4 h. D) Flow cytometry analysis of M2‐phenotype and M1‐phenotype microglia within glioma after treatment. Reproduced with permission.^[^ ^192^ ^]^ Copyright 2021, Wiley‐VCH.

Microglia cells are critical immune cells of the central nervous system (CNS) and serve as brain‐resident macrophages.^[^ ^188^ ^]^ Unlike macrophages derived from peripheral monocytes, microglia originate from yolk‐sac‐derived progenitors and permanently reside in the brain tissue, which play a crucial role in brain development and maintaining homeostasis of CNS.^[^ ^189^ ^]^ Moreover, microglia cells are involved in immunosurveillance and function as the first‐line responder to defense brain damage and exogenous infection. Although largely unknown, accumulating evidence indicated that microglia cells are also involved in the formation of immunosuppressive TME of glioma.^[^ ^190^ ^]^ Resident microglia are recruited to the malignant glioma and instead of initiating the antitumor immunity, they switch to a pro‐tumoral phenotype to suppress antitumor immunity and to support tumor growth, invasion, angiogenesis.^[^ ^191^ ^]^ Due to the shared lineage, microglia and macrophages both share the anti‐tumor phenotype (M1‐like) and pro‐tumoral phenotype (M2‐like). Therefore, microglia cells can serve as a potential target to design biomaterial‐based immunotherapy toward glioma. For example, Gao et al. developed a virus‐mimicking membrane‐coated nucleic acid nanogels Vir‐Gel loaded with therapeutic miRNA (miR155), which aimed to reprogram microglia from pro‐invasive M2 phenotype to antitumor M1 phenotype.^[^ ^192^ ^]^ This Vir‐Gel delivery system was constructed from a miR155‐loaded DNA‐grafted polymer core (nanogel) and then this nanogel was coated with erythrocyte membrane, leading prolonged circulation time. Finally, this membrane‐coated nanogel was further functionalized with two peptides M2pep and HA2. After systemic administration, M2pep could specifically target M2 microglia to enable targeting delivery of miR155‐loaded Vir‐Gel system to microglia. Meanwhile, HA2 peptide, derived from influenza virus, promotes the fusion of erythrocyte membrane with microglia membrane, leading to enhanced internalization of miR155‐loaded Vir‐Gel. Upon escaping from lysosome and entering into cytoplasm, miR155 could be released from miR155‐loaded Vir‐Gel in response to ribonuclease H (RNase H) and remodeled the M2‐like microglioma to M1‐like microglia, leading to reverse of immunosuppressive TME to immunogenic TME (**Figure** **15**). In vitro studies showed that treatment with Vir‐Gel significantly increased the inducible nitric oxide synthase (iNOS) production, a specific marker of M1‐like microglia, compared to other treatments. In contrast, the expression of CD206, a marker of M2‐like microglia, was decreased obviously. Living imaging demonstrated that Vir‐Gel could deliver to glioma site specifically with much higher accumulation after systemic injection. Moreover, treatment with Vir‐Gel prolonged the survival time of glioma‐bearing mice and led to a much higher percentage of M1‐like microglia within glioma TME. All these results indicated the excellent ability of Vir‐Gel to reprogram M2‐like BV‐2 cells to M1‐like phenotype and validated the potential of M2‐like microglia as potential target for designing biomaterial‐based cancer immunotherapies against glioma.

### 6.4 NPs‐Based Targeting Modulation of MDSCs

**Figure 16:**
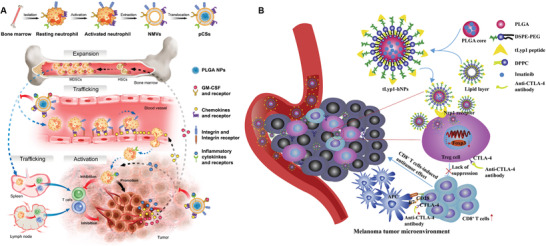
NPs‐based targeted modulation of MDSCs and Treg. A) Schematic illustration of the preparation of pCSs and mechanism of pCSs for disrupting the expansion, trafficking, and activation of MDSCs. Reproduced with permission.^[^ ^196^ ^]^ Copyright 2020, American Chemical Society. B) Schematic illustration of proposed mechanism of tLyp1‐hNPs for targeting Treg cells in the TME. Reproduced with permission.^[^ ^197^ ^]^ Copyright 2018, Elsevier.

MDSCs, an important type of immunosuppressive immune cells, are garnering increasing attention given their critical role in tumor development.^[^ ^193^ ^]^ Generally, MDSCs originate from bone marrow and can be recruited to solid tumors and peripheral lymphoid organs (spleens and LNs), which are identified in various types of cancer, including breast cancer, lung cancer, and hepatocellular carcinoma.^[^ ^193b^ ^]^ Accumulating preclinical and clinical data indicated a negative correlation between high infiltration of MDSCs in the TME with patient prognosis, attributing to that MDSCs can suppress effector T cells and NK cells while expand Tregs.^[^ ^194^ ^]^ Therefore, MDSCs are an important target for enhancing the antitumor immunity. Recent strategies highlighted that MDSCs‐targeted modulation, including the inhibition of MDSCs immunosuppressive activity, differentiate MDSCs into mature cells, blocking the development of MDSCs, or direct MDSCs depletion, are promising for reversing immune tolerance in the TME.^[^ ^195^ ^]^ Li et al. reported a pseudoneutrophil cytokine sponges (denoted pSCs) to disrupt the expansion, recruitment, and activation of ploymorphonuclear MDSCs (PMN‐MDSCs) (**Figure** **16**A).^[^ ^196^ ^]^ Specifically, they developed nanosized pCSs by coating the neutrophil membrane vesicles (NMVs) onto PLGA NPs. The resulting pCSs inherited most membrane receptors from the “parental” neutrophils with a right‐side‐out receptor orientation on the surface, enabling them to act as decoys to absorb and neutralize the corresponding cytokines. In vitro binding assays demonstrated that pCSs markedly absorbed GM‐CSF and CXCL2 in a concentration‐dependent manner. Additionally, treatment with pCSs significantly decreased the concentration of CXCL2 in the circulating blood to an undetectable level in B16F10 tumor‐bearing mice (tumor size ≈200 mm^3^). Time‐lapse intravital microscopy imaging of control tumors without pCSs treatment showed that abundant neutrophils adhered to the endothelial wall within 30 min of excision. In contrast, most neutrophils in mice treated with pCSs were freely flowing in the bloodstream without attaching to inflammatory endothelial cells, suggesting the potential of pCSc to block extravasation of neutrophil driven by chemoattractants. Treatment with pCSs resulted in an obvious inhibition of the MDSCs differentiation as well as of tumor growth B16F10 tumor. Moreover, when combining pCSc with *α*PD1, B16F10 tumor‐bearing mice showed much longer survival compared to that treated with pCSs or *α*PD1 antibody alone.

### 6.5 Targeting Modulation of Treg

Treg cells are an immunosuppressive class of CD4^+^ T cells that possess high CD25 (the interleukin‐2 receptor *α*‐chain) and Foxp3 expression. Treg play a pivotal role in maintaining immunological self‐tolerance by suppressing aberrant immune responses to a broad range self‐antigens or quasi‐self‐antigens with genetic mutations.^[^ ^198^ ^]^ The deficiency or dysfunction of Treg is associated with severe and fatal autoimmune diseases and chronic inflammation in human.^[^ ^199^ ^]^ In healthy condition, there is a balance between Treg cells and other immune cells, required for normal inflammation and tolerance.^[^ ^200^ ^]^ However, this balance is disturbed in the TME because of the infiltration of a large number of Treg cells.^[^ ^201^ ^]^ Most of them are chemo‐attracted to tumor tissues, expanding locally and differentiating into a Treg cell subpopulation that strongly suppresses the activation, expansion, and cytokine production of tumor antigen‐specific effector T cells through multiple mechanisms.^[^ ^199^, ^201^ ^]^ Immunosuppression in TME induced by Treg cells is regarded as a critical mechanism of immune escape and is often related with poor prognosis in a number of cancers, posing a major challenge to cancer immunotherapy.^[^ ^201^, ^202^ ^]^ Given their importance in cancer progression and metastasis, increasing interest are now focusing on targeting the Treg cell therapeutically, such as blocking Treg recruitment into tumor, inhibition of Tregs function with ICB or depleting Treg directly.

Imatinib (IMT), a tyrosine kinase inhibitor that blocks STAT3 and STAT5 signaling, has been shown to lower Treg cell abundance and to impair their immunosuppressive function. However, the poor solubility and cytotoxic effect on normal cells limit the usage of this hydrophobic drug. Neuropilin‐1 (Nrp‐1) is a cell surface transmembrane glycoprotein that interacts with class 3 semaphorins subfamily and members of the VEGF ligand family. It has been reported that the expression of Nrp1 on Treg was correlated with the Foxp3 expression and thus is regarded as promising target candidate. Based on these, Ou et al. developed tLyp1 peptide‐functionalized hybrid NPs loaded with IMT (tLyp1‐hNPs) for targeting Treg cells in the TME (Figure 16B). The tLyp1 peptide belongs to cell‐penetrating peptide which has a C‐terminal R/KXXR/K consensus sequence, also known as the C‐end rule (CendR) motif.^[^ ^203^ ^]^ This kind of peptide was regarded as a substrate with high affinity and specificity for Nrp1 receptor.^[^ ^204^ ^]^ To determine the influence of IMT‐loaded tLyp1‐hNPs on Treg differentiation, an in vitro co‐cultured system consisting of Treg cells and B16 melanoma cells was applied. Treg cell differentiation was relatively higher (39.6%) in the TME than in normal condition (35.2%). After treatment with IMT‐loaded tLyp1‐hNPs, the differentiation reduced to 16.1%, which was much lower than that treated with free IMT (24.6%) and IMT‐loaded hNPs (23.7%). The result suggested that IMT‐loaded tLyp1‐hNPs could efficiently inhibit Treg differentiation. Additionally, the expression of phosphorylated STAT3 (p‐STAT3) and p‐STAT5 in the IMT‐loaded tLyp1‐hNPs group was inhibited to a great extent compared to free IMT, which is in line with the Treg differentiation result. More importantly, melanoma‐bearing mice treated with IMT‐loaded tLyp1‐hNPs combined with *α*CTLA4 showed remarkably synergistic tumor inhibition efficiency compared to control groups. Moreover, a significantly lower percentage of Treg populations in both spleen and tumor as well as higher ratio (*p* < 0.01) of CD8^+^ effector T cells to Treg cells were observed after treatment with IMT‐loaded tLyp1‐hNPs, indicating the enhanced cytotoxic CD8^+^ T cell activation. This study confirmed the notion that targeted inhibition of Treg differentiation is a potential strategy to boost cancer immunotherapy.

### 6.6 NP‐Based Targeting Modulation of CAFs

CAFs refer to a subset of fibroblasts existed in tumor stroma space. CAFs are found in many types of cancer (e.g., breast, colorectal, and ovarian cancer) and often account for a major portion of the tumor stromal cell population.^[^ ^205^ ^]^ Accumulating evidence suggest that CAFs play a predominant role in tumor progression, metastasis, and immune resistance through several mechanisms.^[^ ^206^ ^]^ First, CAFs promote cancer cell proliferation and epithelial cell transformation by secreting high levels of growth factors such as hepatocyte growth factor (HGF), epidermal growth factor (EGF), and insulin‐like growth factor (IGF).^[^ ^207^ ^]^ Second, CAFs can suppress antitumor immunity through secreting various immunosuppressive cytokines, such as IL‐6, IL‐10, TGF‐*β*, and CXCL12.^[^ ^206b^ ^]^ Third, CAFs secret dense ECM components and ECM‐degrading proteases, leading to accelerated ECM renew. These dense ECM leads to restricted T cells infiltration into stroma and prevents effector T cells from physically approaching tumor cells.^[^ ^206^, ^208^ ^]^ Fourth, CAFs are also responsible for the lack of oxygen supply because the dense ECM stiffen the tumor and abnormally squeeze tumor blood vessels to reduce blood supply.^[^ ^206^, ^209^ ^]^ The insufficient oxygen supply along with overstretched oxygen expenditure in tumor further leads to the formation of hypoxia microenvironment, which aggravates the immunosuppressive TME.^[^ ^210^ ^]^ Therefore, depleting CAFs or reversing their functions should be a feasible strategy to suppress tumor metastasis. For example, Zhen et al. developed a NP‐based photoimmunotherapy (nano‐PIT) by conjugating photosensitizer apoferritin (ZnF_16_Pc), a NP protein cage, with a fibroblast‐associated protein (FAP)‐targeted single chain variable fragment (scFv).^[^ ^211^ ^]^ FAP is overexpressed on the surface of CAFs, has been proposed as a potential target. The resulting nanoconjugate, Z@FRT‐scFv, can selectively home to CAFs in tumors after systemic injection. Subsequent photoirradiation led to the depletion of CAFs whereas normal tissues were minimally affected. Moreover, the depletion of CAFs further destructed ECM and decrease CXCL12 secretion, leading to increased infiltration of CTLs and a reverse of immunosuppressive TME to immunogenic TME. In vivo antitumor studies demonstrated that treatment with Z@FRT‐scFv plus photoirradiation significantly suppressed tumor growth and prolonged survival time of tumor‐bearing mice, especially with two dose photoirradiation. Further investigation revealed that treatment with this nano‐PIT could greatly reduced the population of CAFs within TME, leading to improved PIT effect. In a recent study, Huang et al. proposed a Nano‐sapper consisting of a calcium phosphate liposome (CaP) core that co‐loaded with antifibrotic phosphates‐modified *α*‐mangostin (*α*‐M) and plasmid encoding immune‐enhanced cytokine LIGHT (tumor necrosis factor superfamily 14, TNFSF14).^[^ ^212^ ^]^ The CaP was further functionalized with an ECM glycoprotein (tenascin C) targeting peptide (FHK) to fabricate FHK‐pLIGHT@CaMP. *α*‐M, a natural xanthone isolated from the pericarps of mangosteen was selected since it had been reported to reduce liver fibrogenesis without obvious hepatotoxicity.^[^ ^213^ ^]^ LIGHT is a pleiotropic inflammatory cytokine which can promote vascular inflammation and stimulate antitumor immunity through secreting CTLs‐recruiting chemoattractants (CCL13 and CCL21).^[^ ^214^ ^]^ This Nano‐sapper could simultaneously reduce the physical obstacles in TME and recruit CTLs to potentiate immunotherapy against prostate cancer by inactivating abnormal CAFs, destructing ECM density, normalizing the intratumoral vasculatures as well as upregulating the lymphocyte‐recruiting chemoattractants expression. On the one hand, treatment with these Nano‐sapper inactivated CAFs within TME of pancreatic ductal adenocarcinoma (PDAC), leading to downregulated ECM components, such as collagen, fibronectin, and FAP. On the other hand, this Nano‐sapper efficiently normalized the tumor blood vessels. The downregulated ECM components and normalized blood vessels synergistically led to enhanced CTLs infiltration in PDAC, and thus, improved the ICB efficiency when combined with *α*PD1. This study validated the benefits of inactivating CAFs in facilitating CTLs infiltration and enhancing immunotherapeutic effect.

### 6.7 MN‐Based Localized Modulation

**Figure 17:**
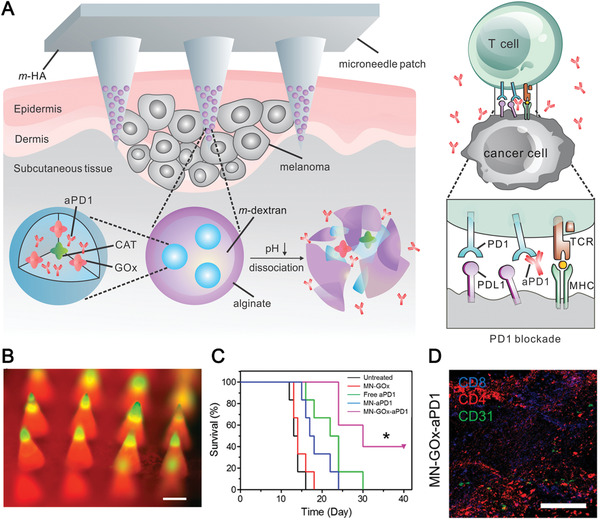
MN‐based localized modulation. A) Schematic diagram of MN patch‐assisted delivery of *α*PD1 for skin cancer treatment. GOx/CAT enzymatic system immobilized inside the NPs by the double‐emulsion method leading to controlled and sustained release of *α*PD1. B) Fluorescence imaging of a representative MN patch that combined *α*PD1‐loaded NPs. C) Kaplan–Meier survival curves for the treated and the control mice (*n* = 8). D) Immunofluorescence staining of tumors showed CD4^+^ T cells and CD8^+^ T cells infiltration post‐treatment with MN‐GOx‐*α*PD1 (scale bar: 100 µm). Reproduced with permission.^[^ ^155^ ^]^ Copyright 2016, American Chemical Society.

Although ICB therapy based on antagonistic antibodies demonstrated treatment benefits in various tumors, their administration routes are mainly relying on i.p injection and thus their therapeutic potential is not fully exploited. To further enhance therapeutic efficacy of ICB antibodies and reduce dose‐limiting side effects, biomaterials enabling localized delivery of ICB antibodies may be a good strategy. As one of promising platform, MN patch has been widely exploited in vaccine delivery as discussed earlier. Recently, the use of MN for delivering ICB antibodies is now receiving increasing attention. For example, Wang et al. developed a self‐degradable MN patch for the sustained delivery of *α*PD1 in a physiologically controllable manner against melanoma (**Figure** **17**A,B).^[^ ^155^ ^]^ The MN is composed of biocompatible hyaluronic acid (HA) integrated with pH‐sensitive dextran NPs that encapsulate *α*PD1 and glucose oxidase (GOx), which converts blood glucose to gluconic acid. Treatment with such MN‐GOx‐*α*PD1 showed a significant and sustained tumor inhibition, with some of tumors even disappeared. They also found that 40% of melanoma‐bearing mice still survived 40 days after treatment with MN‐GOx‐*α*PD1 patch, mainly attributing to the sustained release of *α*PD1 by MNs in the tumor site and enhanced retention of *α*PD1 in TME (Figure 17C). Immunofluorescence staining further revealed that tumor from MN‐GOx‐*α*PD1 treated mice was remarkably infiltrated by both CD8^+^ and CD4^+^ T cells (Figure 17D). Moreover, another study conducted by same group that using this MN patch for combination delivery of *α*PD1 with other immune checkpoint inhibitor, 1‐methyl‐DL‐tryptophan (1‐MT, an inhibitor of IDO), toward local tumor‐specific T cells modulation simultaneously.^[^ ^215^ ^]^ In vivo antitumor study demonstrated that the resulting HA‐NP embedded MN (NP‐HA) could induce robust antitumor immunity to control tumor growth, further suggesting MN patch could be a promising platform for localized delivery of immunomodulatory agents to TME. These studies indicated that MN has great potential to locally deliver immunomodulatory cargoes to TME with higher accumulation and specificity, thus enabling localized modulation of immunosuppressive TME.

### 6.8 Injectable Gel‐Based Localized Modulation

**Figure 18:**
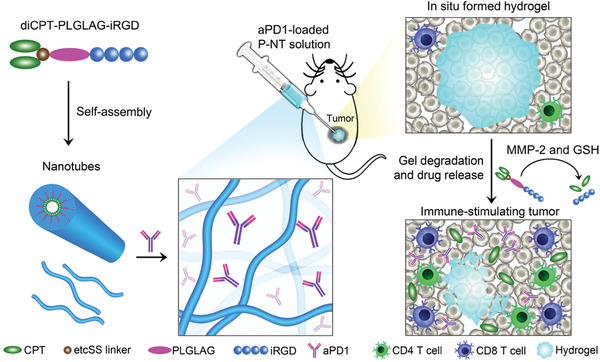
Injectable gel‐based localized modulation. Schematic illustration of preparation of diCPT‐PLGLAG‐iRGD‐based P‐NT hydrogel and its function to attain bioresponsive drug release and TME regulation. Reproduced with permission.^[^ ^156^ ^]^ Copyright 2020, American Association for the Advancement of Science.

Hydrogels are highly appealing drug delivery systems that allow localized delivery of various bioactive cargos, with a potential for controlled and sustainable release.^[^ ^216^ ^]^ To achieve localized modulation, Wang et al. developed a supramolecular prodrug hydrogel for localized delivery of *α*PD1 and camptothecin (CPT, a chemotherapeutic) to synergistically boost the host's immunity against cancer (**Figure** **18**).^[^ ^156^ ^]^ They first synthesized an amphiphilic prodrug, diCPT‐PLGLAG‐iRGD, of which iRGD is peptide known to bind to Nrp‐1 and facilitate tumor interstitial penetration,^[^ ^217^ ^]^ and PLGLAG is a peptide sensitive to MMP‐2. Meanwhile, the two CPT moieties were conjugated with PLGLAG through reducible disulfanyl‐ethyl‐carbonate (etcSS) linker. The diCPT‐PLGLAG‐iRGD can spontaneously assembly into supramolecular nanotubes (P‐NTs), with 10 µm in length in aqueous phase. Furthermore, the adding of PBS or culture medium could induce a rapid and secondary self‐assembly of supramolecular hydrogel. This prodrug hydrogel can be used for delivering *α*PD1 by simply mixing before gelation. In vivo study showed that diCPT‐PLGLAG‐iRGD could rapidly form in situ P‐NT hydrogel within 5 min after s.c. injection and degraded very slow, with 78% degraded within 45 days. The release study showed that *α*PD1‐encapsulating P‐NT hydrogel could significantly extend the local retention and release of *α*PD1 after intratumoral injection compared to *α*PD1 solution. More importantly, P‐NT‐*α*PD1 could enable sustained release of CPT to kill tumor cells and sustained release of *α*PD1 to block PD1 on tumor‐specific CTL. Thus, P‐NT‐*α*PD1 synergistically induced strong antitumor immunity and significantly suppressed the tumor growth after intratumoral injection compared to other treatments. Moreover, treatment with P‐NT‐*α*PD1 could also induced a durable memory immunity against tumor rechallenge. In a following study by same group, they used similar strategy that CDA (a STING agonist) was electronically complexed with P‐NT hydrogel (CDA‐NT).^[^ ^218^ ^]^ It has been reported that the activation of STING pathway in tumor cells could stimulate innate and adaptive immunity within TME.^[^ ^219^ ^]^ In this study, they conjugated two CPT moieties with iRGD directly through etcSS linker to synthesize diCPT‐iRGD. In vivo studies demonstrated that CDA‐NT could also form in situ supramolecular hydrogel, which enabled localized delivery of CDA and CPT to tumor cells with substantially enhanced retention. As expected, CDA‐NT could stimulate strong localized antitumor immunity via a synergistic effect of CDA and CPT to control tumor growth as well as induced durable memory immunity. These studies indicate the great potential of supramolecular hydrogel for localized modulation of specific cells within TME.

### 6.9 Implantable Scaffold‐Based Localized Modulation

**Figure 19:**
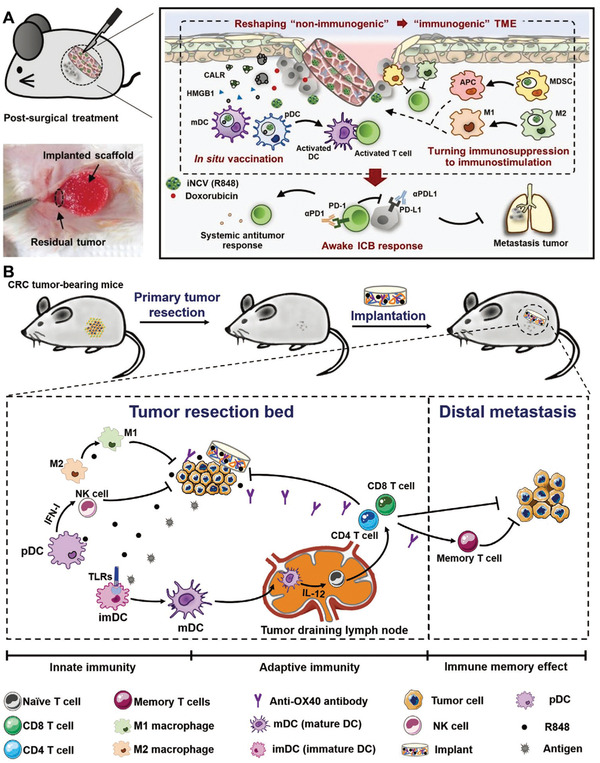
Implantable scaffold‐based localized modulation. A) Schematic diagram of the design of scaffold combo co‐loaded with iNCVs, DOX, and *α*PDL1/*α*PD1antibodies to reverse nonimmunogenic TME into immunogenic TME. Reproduced with permission.^[^ ^220^ ^]^ Copyright 2019, Wiley‐VCH. B) Schematic illustration of the biopolymer‐derived BI(R848+*α*OX40) scaffold for preventing CRC postoperative tumor relapse and metastasis by eliciting innate and adaptive immunity and immune memory effect. Reproduced with permission.^[^ ^221^ ^]^ Copyright 2020, Wiley‐VCH.

As aforementioned, implantable scaffold can also realize localized delivery and sustained release of encapsulated cargoes, which has been widely used for cancer immunotherapy. For example, Pheungkham et al. developed a designer scaffold co‐loaded with R848‐encapsulated nanoconverters (iNCV) and DOX, which can be implanted into a post‐surgical model for spatiotemporal reverse of nonimmunogenic TME into immunogenic TME (**Figure** **19**A).^[^ ^220^ ^]^ On the one hand, iNCV could not only activate recruited APCs and induce antigen‐specific T cells, but also polarize TAMs and MDSCs into APCs with antitumor phenotypes. On the other hand, DOX, a chemotherapeutic, could induce tumor cells apoptosis to release self‐antigen source, leading to the induction of a host antitumor immune response through the ICD effect. These functions were validated by in vitro studies, suggesting the effective cancer vaccine and immunomodulatory function of this in situ iNCV and DOX‐loaded scaffold (Combo). They further evaluated the postoperative treatment efficiency of this scaffold in advanced 4T1 breast tumor model. Antitumor study showed that treatment with iNCV and DOX‐loaded scaffold significantly suppressed the tumor recurrence and metastasis, resulting in prolonged survival time. Meanwhile, owing to the controlled release behavior, treatment with this combo scaffold showed no significant toxicity in mice compared to treatment with equivalent iNCV, DOX, or physical mixture of iNCV and DOX. Further investigation demonstrated that treatment with Combo scaffold led a significantly enhanced infiltration of effector immune cells while decreased MDSCs. These findings were further confirmed by the cytokines release profiles, of which proinflammatory cytokines (IL‐6, IL‐12, IFN‐*γ*) were upregulated while immunosuppressive cytokine (IL‐10) was downregulated, suggesting Combo scaffold could efficiently convert nonimmunogenic TME into immunogenic TME. Moreover, when combining *α*PD1 or *α*PD‐L1 into this Combo scaffold, the antitumor therapeutic efficiencies were further improved in both postsurgical 4T1 breast tumor model and TC1 cervical tumor model. In a more recent study, Ji et al. developed a biopolymer‐derived implantable scaffold loaded with R848 and *α*OX40 (BI(R848 + *α*OX40)) for post‐surgical colorectal cancer (CRC) immunotherapy (Figure 19B).^[^ ^221^ ^]^ *α*OX40 is an antibody proven to activate T cells while inhibit Treg differentiation as well as induce subsequent memory immunity.^[^ ^222^ ^]^ After s.c. implantation, this biopolymer scaffold could retain at implanted site for at least 21 days, which enabled a sustained release of the encapsulated R848 and *α*OX40 and thus persistent stimulation of the surrounding immune cells. In vivo antitumor studies exhibited that treatment with BI(R848 + *α*OX40) completely eradicated the residual tumor post‐surgery in all the mice and efficiently inhibit tumor relapses in 150 days. Moreover, treatment with BI(R848 + *α*OX40) induced a tumor‐specific memory immunity that could prevent tumor re‐challenge at distal site post‐surgery. Immunological analysis demonstrated that BI(R848 + *α*OX40) could induce innate immunity and adaptive immunity sequentially, contributing to the promising antitumor immunotherapy effect.

Considering the immunomodulatory functions of different cancer immunotherapies toward APCs, T cells and cells‐within TME are taking place in different locations that are histologically distinct, the delivery methods used for different immunotherapies may be different in order to achieve optimal delivery efficiency and immunomodulatory effect. Owing to customizable and tunable features of biomaterials, cancer immunotherapy based on different biomaterials meeting different delivery requirements can be pursued. Because APCs mainly existed in lymphoid organs (LNs, spleen, and skin), APCs‐specific cancer vaccines thus require efficient delivery to these lymphoid organs, which can be achieved by either interstitial administration or i.v. administration. Interstitial administrations (i.t., s.c., i.m.) have been widely used as the delivery methods of MN/hydrogel/macroparticle‐based vaccines or nanovaccines for delivering cancer vaccines to LNs‐resident and skin‐resident APCs. This is due to that there are abundant lymphatic vessels, which ensure effective interstitial trafficking. However, i.v. administration of nanovaccine is only applied for APCs within spleen where plenty of blood vessels exist. Similar to APCs, targeting modulation of cells within TME can also be realized by localized and systemic administrations. Systemic administration is only suitable for NPs‐based immunotherapies and involves two major mechanisms, EPR‐mediated passive targeting and ligand‐mediated active targeting. By contrast, localized administration is mainly referring to these biomaterials which can deliver immunotherapies directly to TME without going through systemic circulation, such as MN, hydrogel, implantable scaffold. It should be noted that localized administration is more suitable for solid tumor that are accessible while may not be suitable for metastatic tumor and haematological malignancy. With regard to T cells, they can be found in lymphoid organs, TME as well as circulatory bloodstream. Therefore, the administration methods used for APCs modulation and TME‐resident cells modulation are also suitable for T cells modulation. Generally, systemic administration is suitable for NPs‐based immunotherapies while localized or interstitial administration is suitable for MN/hydrogel/implantable scaffold‐based immunotherapies. Unlike in vivo T cells modulation that requires delivery methods, biomaterials‐based aAPCs for T cells expansion and T cells engineering ex vivo does not require delivery methods. Taking together, understanding the different requirements by different delivery methods may be beneficial for designing more effective and specific biomaterials‐based immunotherapies.

## 7 Challenge and Prospective

In conclusion, although cancer immunotherapy can yield robust immune response, the percentage of patients who benefit from this treatment remain modest. Biomaterials represent promising tool to overcome the challenges that faced by the current immunotherapy, which have demonstrated improved delivery efficiency and enhanced antitumor efficacy whereas decreased side‐effects compared to free immunotherapy alone. Biomaterials are being explored for the development of prophylactic and therapeutic cancer vaccine, ex vivo expansion and modulation of T cells, in situ engineering of T cells, and targeting modulation of immunosuppressive TME‐resident tumor cells or immune cells. However, significant challenges still exist to achieve broad clinical efficacy with immune cell or tumor cell targeting biomaterials, including identification of tumor‐specific antigens, insufficient CTL infiltration, variation of checkpoint expression, and clinical translation.

Identification of TAAs has been considered as a promising strategy for the development of tumor‐specific vaccines.^[^ ^21c^ ^]^ Most TAAs that currently used in both clinical and preclinical studies are tumor‐self antigens, which are normally expressed in healthy cells whereas overexpressed in tumor cells. This strategy has led to the successful screen and discovery of a variety of TAAs, including HER‐2 (a breast cancer‐associated antigen),^[^ ^223^ ^]^ MAGE‐1 (a melanoma‐associated antigen)^[^ ^224^ ^]^ and NY‐EOS‐1 (a cancer‐testis antigen). However, the clinical benefits from patients who received these TAAs‐based vaccines reflected variable and limited, which were mainly attributed to several factors: i) tumor cells undergo constant evolution with somatic mutation, leading to the generation of many other TAAs with distinctive phenotypes. As a consequence, the identified tumor‐self TAAs with certain phenotype are only a small fraction of these TAAs and vary between patients, which may not be able to induce effective and long‐lasting antitumor immunity; ii) owing to that TAAs are also expressed in healthy tissue, the antitumor immunity desired for these TAAs‐overexpressed tumor cells may also attack normal tissues despite with the help of targeting biomaterials, resulting in off‐target autoimmune effects; iii) TAAs‐based vaccines may be subject to central tolerance governed by T cells, resulting in low immunogenicity.^[^ ^4^, ^225^ ^]^ Therefore, the identification of TAAs with more specificity may be good candidate for developing personalized cancer vaccine. To date, two major strategies have been developed: one is the use of autologous tumor cells and the other is synthetic neoantigen predicted by computerized algorithms.^[^ ^225^, ^226^ ^]^ On the one hand, vaccines based on autologous tumor cells and engineered cell components that prepared by ex vivo treatment or ICD contain almost all TAAs, which are easy manufacturing. However, it should be noted that the use of tumor cell lysate could increase the complexity of clinical translation. On the other hand, the next‐generation sequencing offers the opportunity for comprehensive mapping of all mutations in a cancer patient, which has been used for mutated gene identification and corresponding neoantigen prediction. Subsequently, these predicted neoantigens are further evaluated and confirmed through experimental immunogenic screening, such as mass spectrometry.^[^ ^227^ ^]^ Thus, computational and experimental pipelines have been considered as a major method for identifying personalized neoantigens in real‐time.^[^ ^226^ ^]^ Compared to identified tumor‐self TAAs, neoantigens are more endogenous and close to naturally existed antigens in tumor, which have been validated with higher immunogenicity in preclinical and clinical studies. Additionally, the number and type of neoantigens are unique to each lesion, which might necessitate a personalized immunotherapeutic approach.^[^ ^225a^ ^]^ By taking advantage of biomaterials, synthetic neoantigen or tumor cell lysate‐loaded vaccine may elicit more specific and effective antitumor immunity.

Recently, mRNA therapy has emerged as a promising therapeutic agent to prevent and treat various diseases.^[^ ^228^ ^]^ Particularly, the success of mRNA vaccines developed by Moderna and BioTech/Pfizer against COVID‐19 pandemic has brought the mRNA therapy into spotlight of the scientific community, which makes a milestone for mRNA therapy.^[^ ^229^ ^]^ Since then, the development of mRNA vaccine to prevent and treat various diseases has been greatly promoted. In addition to virus infection, mRNA has also been widely explored as neoantigen for designing personalized cancer vaccines. mRNA vaccines possess multiple superiorities over peptide or protein vaccines, including high compatibility, safe and relatively simple manufacturing, non‐integrative properties, predictable and consistent protein expression kinetics, as well as the flexibility to encode a wide variety of peptide and protein structures.^[^ ^228^, ^230^ ^]^ mRNA vaccines also show compatible advantages over DNA vaccines, such as mRNA undergoes a rapid and transient expression of encoded proteins in cytoplasm without the need of nuclear entry. Thus, mRNA avoids inserting into the host genome which may cause detrimental insertational mutagenesis.^[^ ^228^, ^231^ ^]^ To date, a plenty of clinical trials have explored mRNA vaccines for cancer immunotherapy over the past decades, with more than 600 cases are ongoing according to ClinicalTrials.gov. However, their widespread applications and successful clinical translation have been limited by several challenges, such as perceived instability, susceptibility to degradation, rapid clearance by immune system, insufficient intracellular expression of protein, as well as inadequate antigen loading and maturation of APCs.^[^ ^232^ ^]^ To function in vivo, mRNA requires safe, effective and stable delivery systems that protect mRNA from degradation and allow cellular uptake and mRNA release. Viral vectors have been used as mRNA carriers but may suffer from their potential immunologic side effects and toxicity as well as the vector‐size limitation.^[^ ^233^ ^]^ Non‐viral strategies such as electroporation,^[^ ^234^ ^]^ gene gun^[^ ^235^ ^]^ and sonoporation^[^ ^236^ ^]^ have been more thoroughly investigated as mRNA delivery system. However, these strategies require ex vivo manipulation of cells, which are laborious and high‐cost.^[^ ^232b^ ^]^ Further refinement of in vivo mRNA delivery modalities is therefore essential for its development as therapeutic tool. Fortunately, various nanocarriers have been established and exhibited a promising foundation for the development of mRNA delivery platform, such as protamine‐mRNA complex, LNPs, polycation micelles, polymer‐lipid hybrid NPs, and gold NPs.^[^ ^232b^ ^]^ Among these, ionizable LNPs have received significant attention due to their success in two COVID‐19 vaccines development: BNT162b^[^ ^237^ ^]^ and mRNA1273.^[^ ^238^ ^]^ Moreover, many other mRNA‐lipid NPs formulations have been developed and are currently under clinical investigation as well as preclinical studies for prevention of a variety of diseases, including cancer.^[^ ^239^ ^]^ To ensure successful clinical translation of mRNA‐lipid NPs formulations, a better and comprehensive understanding of their rational design, possible interaction with physiological barriers, and potential administration route is required. Meanwhile, good manufacturing practice, stability, storage and safety should also be considered.

One of the most representative hallmarks of TME is abnormal vasculature, resulted from rapid angiogenesis. These abnormal vasculatures are often characterized by discontinuous endothelial junctions and abnormally large pore size, and thus blood and fluid are easy to penetrate through the vascular pore into tumor interstitium, leading to increased interstitial fluid pressure (IFP).^[^ ^240^ ^]^ Meanwhile, the proliferation of CAFs, one key component of TME, contributed to the dense ECM, which further compress the vasculature. The increased IFP along with dense ECM not only restricts the penetration of nanomedicine from blood into tumor interstitium but also hindered the infiltration of activated T lymphocytes into TME. Meanwhile, these pathophysiological features of TME also limit the blood flow and thus reduce the oxygen supply, leading to hypoxia.^[^ ^210^ ^]^ It has been widely documented that hypoxia plays a critical role in inducing immunosuppressive TME and thereby compromise efficiency of immunotherapy. Taking together, the TME is a major cause of the failure of both nanomedicines and immunotherapies. Therefore, normalizing TME may offer the opportunities to simultaneously improve the perfusion of nanomedicines and alleviate hypoxia‐induced immunosuppression in the TME. One strategy is to normalize the tumor vasculature by directly or indirectly using antiangiogenic therapies (AATs) so that it becomes more morphologically and functionally similar to the vasculature of normal tissue.^[^ ^240^ ^]^ The other strategy is to reprogram CAFs to reduce the ECM levels and thus decompress the blood vessels, leading to an improved perfusion and oxygen supply.^[^ ^241^ ^]^ Accumulating evidences suggested that improvement in tumor perfusion and alleviation of hypoxia increase antitumor efficiency of several cancer immunotherapies, such as cancer vaccine,^[^ ^242^ ^]^ ICB,^[^ ^243^ ^]^ and ACT.^[^ ^244^ ^]^ Therefore, synergistic administration of TME‐normalizing agents with cancer immunotherapies based on biomaterials may further enhance CTLs infiltration and thus improve the antitumor immunity. For example, Feng et al. developed a dual targeting gene delivery system loading with two therapeutic plasmids DNA pshVEGF‐A and pshPD‐L1, termed as RHVP NPs.^[^ ^245^ ^]^ After i.v. injection, RHVP NPs could target tumor cells through the interaction between HA and CD44. Then, therapeutic pshPD‐L1 could downregulate PD‐L1 expression to reduce the adaptive resistance and therapeutic pshVEGF‐A could normalize the abnormal vasculature to alleviate hypoxia, leading to a synergistic reprogram of immunosuppressive TME to immunostimulatory TME. Therefore, treatment with RHVP NPs significantly inhibited the tumor progression and alleviated the pulmonary metastasis. As discussed in Section 6.5, biomaterials‐based targeting modulation of CAFs represents another promising strategy to reprogram dense ECM and to alleviate hypoxia, leading to improved cancer immunotherapy.

With the advance of material science and delivery technologies, more specific and efficient modulation toward specific immune cells or tumor cells can be pursed. Despite intensive investigation on biomaterials‐based immunotherapies, only a very limited number of products move forward from bench to bedside application (**Table** **2**). How to increase the clinical translation potential of biomaterials‐based immunotherapies is thereby the major challenge when designing biomaterial‐based immunotherapies. Several critical prerequisites should be met before biomaterial‐based immunotherapies can be widely commercialized, including high safety, scale‐up manufacturing, batch‐to‐batch quality control, and long‐term stability. For safety concern, one strategy to use biocompatible synthesized biomaterials with low immunogenicity and toxicity, such as liposome, LNPs, albumin NP, biodegradable polymeric NPs, macroparticles or MN that fabricated using FDA‐approved materials. A typical example is the PLGA‐based scaffold vaccine, termed as WDVAX (NCT01353089) developed by Mooney and colleagues, which is now being investigated in a phase I clinical trial for stage IV melanoma and has been licensed to Novartis for commercial use. Another example is the use of LNPs for developing mRNA cancer vaccines, mRNA‐2752 (NCT03739931),^[^ ^246^ ^]^ and mRNA‐2416 (NCT03323398). Moreover, naturally derived materials, such as extracellular vesicles, hyaluronic acid, chitosan, collagen, are also widely used for developing cancer immunotherapies. One sample is to load DC‐derived exosomes with antigenic peptides to construct exosome‐based vaccines (Dex2) for treating advanced NSCLC, which is now completed in Phase II clinical trials (NCT01159288). However, progression free survival is observed during the Phase II study. Regardless of synthesized or naturally derived, these biomaterials that hardly or minimally interact with host system are more feasible for clinical translation. For manufacture and quality control, although deliciated design may ensure multiple functionalities, it also complicates the produce process. In comparison, simplified design is more likely to achieve scale‐up manufacturing with more controllable quality. Also, it should be noted that some naturally derived biomaterials are difficult to achieve massive production, standard isolation, long‐term maintenance of stability, and quality compared to synthesized biomaterials. Therefore, the desirable functionalities and scale‐up manufacturing and quality control should be well balanced, and more efforts are needed to explore novel biomaterials that possess clinical translation potential.

**Table 2:** Summary of the biomaterial‐based cancer immunotherapies under clinical trial

Name^i)^	Materials	Component	Cancer	Clinical trial stage
Nanovaccine				
Dex2	DC‐derived exosome	MAGE, NY‐ESO‐1, MART‐1 peptides	Advanced ^a)^NSCLC	Phase II, completed NCT01159288)
DPX‐Survivac	Liposome	Survivin, cyclophosphamide, epacadostat	Recurrent ovarian cancers	Phase 1 ongoing NCT02785250)
	Liposome	Survivin, cyclophosphamide	Recurrent Survivin‐expressing diffuse large B‐cell lymphoma	Phase II ongoing NCT02323230)
			dvanced stage of ovarian, fallopian or peritoneal cancer	Phase I, ongoing NCT033325576)
	Liposome	Survivin, cyclophosphamide, and *α*PD1	ARecurrent diffuse large B‐cell lymphoma	Phase II ongoing NCT03349450)
			Advanced ovarian, primary peritoneal or fallopian tube cancers	Phase II, ongoing NCT03029403)
DPX‐0797	Liposome	7 tumor‐specific HLA‐A2‐restricted peptides, a universal T helper peptide, a polynucleotide adjuvant	Advanced ovarian, breast, and prostate cancers	Phase I, completed NCT01095848)
Dribbles	^b)^TDA	N/A	Advanced NSCLC	Phase I, ongoing NCT03057340)
	TDA	HPV vaccine, R848	Advanced prostate cancers	Phase I, ongoing NCT02234921)
	TDA	HPV vaccine, R848 or GM‐CSF	Stage III NSCLC	Phase II, ongoing NCT01909752)
IMF‐001	NP complex	NY‐ESO‐1 protein, cholesteryl pullulan	Recurrent esophageal cancers	Phase I, completed NCT01003808)
Lipo‐MERIT	Liposome	RNA antigen	Stage III‐IV melanoma	Phase 1 ongoing NCT02410733)
mRNA‐2752	LNP	mRNA encoding human OX40L, IL‐23, and IL‐36*γ*	^c)^TNBC, ^d)^HNSCC, Non‐Hodgkin's, urothelial cancer, melanoma, NSCLC lymphoma	Phase I, ongoing NCT03739931)
mRNA‐2416	LNP	mRNA encoding human OX40L	Advanced malignancies	Phase I, ongoing NCT03323398)
ONT‐10	Liposome	Synthetic glycolipopeptide, PET lipid A adjuvant	Stage III‐IV solid tumors	Phase I, completed NCT01556789)
	Liposome	Synthetic glycolipopeptide, PET lipid A adjuvant, Varlilumab	Advanced ovarian and breast cancer	Phase I, completed NCT02270372)
PAN‐301‐1	NP	HAAA peptide	Recurrent prostate cancer	Phase I, ongoing NCT03120832)
PRECIOUS‐01	PLGA NP	NY‐ESO‐1 peptide, theritolceramide	NY‐ESO‐1‐positive cancers	Phase I, ongoing NCT04751786)
W_ova1	Liposome	3 ovarian cancer mRNA	Ovarian cancer	Phase I, ongoing NCT04163094)
ZYC300	PLG microparticles	Plasmid DNA, CYP1B1	Advanced stage, progressive cancer	Phase I, completed NCT00381173)
N/A	Liposome	^e)^LAMP mRNA	^f)^MGMT unmethylated ^g)^GBM	Phase I, ongoing NCT04573140)
Scaffold‐based vaccine				
WDVAX	Macroporous PLG scaffold	Tumor lysates, GM‐CSF, CpG	Metastatic melanomas	Phase I, ongoing (NCT01753089)
Hydrogel‐based vaccine				
N/A	Hydrogel	GM‐CSF plus ^h)^BCG	Unresectable colorectal liver metastasis post‐radiofrequency	Phase I, ongoing (NCT04062721)

^a)^ NSCLC: non‐small cell lung cells;

^b)^ TDA: tumor‐derived autophagosomes;

^c)^ TNBC: triple‐negative breast cancer;

^d)^ HNSCC: head and neck squamous cell carcinoma;

^e)^ LAMP: lysosomal associated membrane protein;

^f)^ MGMT: methylation of O6‐methyguanine‐DNA methytransferase;

^g)^ GBM: glioblastoma multiform;

^h)^ BCG: bacteria protein derivatives can be TLR or nucleotide‐binding oligomerization domain 2 (NOD2);

^i)^ More abbreviations can be found in the Supporting Information.

## Conflict of Interest

The authors declare no conflict of interest.

## Supporting information

Supporting Information
