# Supplementary material for: Advanced Biomaterials for Cell‐Specific Modulation and Restore of Cancer Immunotherapy
Source: Adv Sci (Weinh). 2022 Mar 27;9(16):2200027. doi: 10.1002/advs.202200027 (PMC9165523; doi:10.1002/advs.202200027)
Supplement: Supplementary file 1 — Supporting Information [file ADVS-9-2200027-s001.pdf]

ADVANCED SCIENCE
Open Access

Supporting Information

for *Adv. Sci.*, DOI 10.1002/advs.202200027

Advanced Biomaterials for Cell-Specific Modulation and Restore of Cancer Immunotherapy

Shaobo Ruan, Yuanyu Huang, Mei He and Huile Gao*

Supporting Information

Advanced biomaterials for cell-specific modulation and restore of cancer immunotherapy

Shaobo Ruan, Yuanyu Huang, Mei He, and Huile Gao*

Table S1. Summary of the full title to abbreviation.

Abbreviation	Full title
AATs	antiangiogenic therapies
ALDH	aldehyde dehydrogenase
APCs	antigen presenting cells
aAPCs	artificial antigen presenting cells
AuNPs	gold nanoparticles
Bcl-2	B cell lymphoma 2
CAFs	cancer-associated fibroblasts
CAR-T	chimeric antigen receptor T-cell
CD	β-Cyclodextrin
CDX	humanized cell line-derived xenograft
cGAS	cyclic GMP-AMP synthase
Clec9A	C-type lectin domain containing 9A
CLRs	C-type lectin receptors
CM	charge-group modified
CMV	cell membrane vesicle
CpG	cytosine-phosphodiester-guanine
CPT	camptothecin
CRC	colorectal cancer
CSC	cancer stem cells
CSF1R	colony stimulating factor 1 receptor
CSS	cysteine-serine-serine
CTLA4	Cytotoxic T lymphocyte antigen 4
CTLs	cytotoxic T lymphocytes
DCs	dendritic cells
DC-SIGN	dendritic cell-specific ICAM3-grabbing nonintegrin
Dectin-1	DC-associated C-type lectin-1
DINP	dual immunotherapy nanoparticle
DOX	doxorubicin
ECM	extracellular matrix
EGF	epidermal growth factor
EPR	enhanced permeability and retention
FcRs	Fc receptors
GBM	glioblastoma multiform
GM-CSF	granulocyte macrophage colony stimulating factor

GOx	glucose oxidase
T_H1	helper T cell 1
HCC	hepatocellular carcinoma
HCQ	hydroxychloroquine
HGF	hepatocyte growth factor
HNSCC	head and neck squamous cell carcinoma
HSP	heat shock proteins
ICB	immune checkpoint blockade
ICD	immunogenic cell death
IDO	indoleamine 2,3-dioxygenase
i.d.	intradermal
IFN-α	interferon α
IFP	interstitial fluid pressure
Ig	immunoglobulin
IGF	insulin-like growth factor
IL	interleukin
i.l.	ipsilateral
ILCs	innate lymphoid cells
i.m.	intramuscular
imDCs	immature dendritic cells
IMQ	imiquimod
IMT	Imatinib
iNOS	inducible nitric oxide synthase
irAEs	immune related adverse effects
i.v.	intravenous
KLRG1	killer-cell lectin-like receptor G1
Kyn	kynurenine
LAG-3	lymphocyte activation gene 3
LAMP	lysosomal associated membrane protein
LCs	Langerhans cells
LN	lymph node
LNPs	ionizable lipid nanoparticles
LOX-1	Lectin-like oxidized low-density lipoprotein receptor 1
LP	long peptide
mAbs	monoclonal antibodies
MAN	mannose
mDCs	mature dendritic cells
MDSCs	myeloid-derived suppressor cells
MET	metformin
MGMT	methylation of O6-methyguanine-DNA methytransferase
MHC class I	major histocompatibility complex class I
MHC class II	major histocompatibility complex class I
MMP-2	matrix metalloproteinase-2

MNs	microneedles
MPLA	monophosphoryl lipid A
MPS	mononuclear phagocytic system
MR	mannose receptor
mRNA	message RNA
MSR	mesoporous silica rod
1-MT	1-methyl-DL-tryptophan
MyD88	myeloid differentiation primary-response gene 88
NHS-SS-NHS	disulphide-containing bis-N-hydroxy succinimide
NIR	near-infrared light
NP	nanoparticle
Nrp-1	Neuropilin-1
NSCLC	non-small cell lung cells
OVA	ovalbumin
OXA	oxaliplatin
OX40	necrosis factor receptor superfamily member 4
PAE	poly(β-amino ester)
PAMAM	polyamidoamine
PD1	programmed cell death 1
PD-L1	programmed cell death ligand 1
PDPA	poly(diisopropanolamino ethyl methacrylate-co-hydroxyethyl methacrylate)
PDT	photodynamic therapy
PDX	patient-derived xenograft
PEG	poly(ethylene-glycol)
PLA	poly(lactic acid)
PLGA	poly(lactide-co-glycolide)
PLGA	poly(lactic-co-glycolic acid)
PLL	poly-l-(lysine)
pMHC	peptide-major histocompatibility complex
PPa	pheophorbide A
pSCs	pseudoneutrophil cytokine sponges
RBCs	red blood cells
RNAi	RNA interference
ROS	reactive oxygen species
RP	red pulp
R848	resiquimod
SEM	Scanning electron microscope
sHDL	synthetic high-density lipoprotein
SNP	self-assembling nanovaccine platform
SPIONs	superparamagnetic iron oxide NPs
SR-B1	scavenger receptor class B1

STAT3	signal transducer and activator of transcription 3
STING	stimulator of interferon genes
s.c.	subcutaneous
TAA	tumor associated antigen
TAMs	tumor-associated macrophages
TAMM	TAM-derived membrane
TCR	T cell receptor
TDLNs	tumor-draining lymph nodes
TGF-β	transforming growth factor β
TIM-3	T cell immunoglobulin and mucin domain-containing protein 3
TME	tumor microenvironment
TMZ	temozolomide
Treg	regulatory T cells
TRIF	TIR-domain-containing adaptor protein inducing INF-β
Trp	tryptophan
VEGF	vesicular endothelial growth factor
WP	white pulp
